# First eDNA-based monitoring of marine vertebrates in the urban marine ecosystem of Abu Dhabi: Mangrove and seagrass beds as biodiversity reservoirs?

**DOI:** 10.1371/journal.pone.0352249

**Published:** 2026-08-04

**Authors:** Jean-Luc Jung, Rachel Haderlé, Karen Cosnier, Mohamed Al Musallami, Sara Al Mehairbi, Ponpandi Perumal, Sultan Al Hammadi, Alice Valentini, Visotheary Ung, Christophe Prazuck, Clio Chaveneau, Olivier Adam

**Affiliations:** 1 Ocean Institute, SAFIR, Sorbonne University Abu Dhabi, United Arab Emirates; 2 Institut de Systématique, Évolution, Biodiversité, ISYEB, Muséum National d’Histoire Naturelle, CNRS, Sorbonne Université, EPHE-PSL, Université des Antilles, Paris, France; 3 Marine Station of Dinard, Museum National d’Histoire Naturelle, Dinard, France; 4 Environment Agency, Abu Dhabi, United Arab Emirates; 5 SPYGEN, Le Bourget du Lac, France; Charles University: Univerzita Karlova, CZECHIA

## Abstract

The Arabian Gulf is a semi-enclosed sea with shallow waters. Its marine fauna must cope with extreme and recurring environmental conditions (high summer temperatures, hypersalinity, hypoxia). In addition, local human activities are very significant and have a marked impact on coastal areas, particularly in urban marine ecosystems (UME). Taken together, the Arabian Gulf’s low species richness, its exposure to multiple human-induced stressors, and the proximity of many taxa to their environmental tolerance limits create a particularly concerning situation. Monitoring marine wildlife must be a priority in this context, particularly with respect to marine vertebrates, difficult to study using integrative approaches. The present study aimed to generate relevant data on marine vertebrate biodiversity in the UME of Abu Dhabi, and to provide evidence-based information to inform conservation policy. In addition, investigating marine biodiversity in the world’s warmest sea in summer, the Arabian Gulf, will provide us with information of general interest in terms of the adaptation and evolution of marine fauna in the current period of global climate change. We conducted the first environmental DNA (eDNA)-based survey of marine vertebrate diversity in Abu Dhabi’s UME. Using a standardized process of water sampling, eDNA metabarcoding using a  ~ 97 bp fragment of the mitochondrial 12S rRNA gene as barcode, and taxonomic assignment, we studied vertebrate biodiversity at four urban sites, including a marina, a newly developed area, a seagrass bed, and a protected urban mangrove. We identified 83 distinct taxa, including five birds, two elasmobranchs, one marine mammal, and 75 teleosts. The specific taxonomic richness did not vary significantly between sites. In contrast, phylogenetic diversity metrics revealed lower diversity than expected for the newly developed area, while the mangrove and, to a lesser extent, the seagrass bed exhibited a phylogenetic structure consistent with their species richness. This approach provides a powerful, non-invasive and integrative framework for long-term monitoring and supports evidence-based conservation planning for marine biodiversity in the Arabian Gulf.

## Introduction

Abu Dhabi, the capital of the United Arab Emirates (UAE), lies on a low-lying island in the Arabian Gulf, a shallow, semi-enclosed marginal sea forming part of the Arabian Sea ecoregion within the tropical Indo-Pacific realm [1,2]. The Gulf extends 990 km in length and 56–370 km in width, with a mean depth of 36 m. It is subject to extreme physicochemical conditions, including high temperatures, hypoxia, and salinity [3–8], which have shaped unique coastal and marine ecosystems, notably mangroves, seagrass beds, and coral reefs, often characterized by low species diversity [9–11]. Notably, UAE coral reefs are globally recognized for their tolerance to high temperature and salinity, partly due to their association with the thermotolerant symbiont *Symbiodinium thermophilum* [9–13]. Such conditions and adaptations make the UAE a natural laboratory for studying the potential impacts of elevated ocean temperatures on diverse marine ecosystems [14] and species [15,16].

Despite their resilience, Arabian Gulf marine habitats face increasing anthropogenic pressures, including rapid coastal development, oil extraction, dredging, desalination, and overfishing [17–19]. Approximately 70% of Gulf coral reefs have been lost in recent decades, accompanied by declines in fish species richness [20]. Climate projections suggest that by 2090, local extinction rates for conservation-priority species could reach up to 35% of 2010 levels, with the greatest losses expected in southwestern Gulf waters [6]. This vulnerability is amplified by the Gulf’s naturally low species richness compared to adjacent regions, its exposure to multiple human-induced stressors, and the proximity of many taxa to their environmental tolerance limits [21,22]. As a result, the Gulf coastal waters are both biologically valuable and ecologically fragile, highlighting the urgent need for various robust biodiversity monitorings to guide conservation and management strategies.

The waters off the coast of the Emirate of Abu Dhabi are home to six major protected areas, among them the region’s largest marine biosphere reserve, the Marawah Marine Biosphere Reserve [23]. Over the past two decades, several studies have examined marine biodiversity in the Arabian Gulf and the UAE [14,24,25]. However, many gray areas still need to be explored in terms of marine biodiversity in the waters of the Arabian Gulf, such as marine vertebrate biodiversity in anthropized coastal areas [17].

Abu Dhabi protects and enhances coastal water quality through monitoring, advanced wastewater treatment, marine conservation, and public engagement [26]. The Environment Agency, Abu Dhabi (EAD), leads these efforts for instance with monitoring networks [7]. Abu Dhabi exemplifies an Urban Marine Ecosystem (UME), where coastal development, through chemical and noise pollution, resource exploitation, coastal and underwater construction and ocean sprawl, is thought to alter habitats, reduce foundation species, favor opportunistic and non-indigenous taxa, and impose selective pressures that promote phenotypic plasticity and local adaptation [27]. Monitoring biodiversity in UMEs has become a major concern, technically difficult to implement, but required to understand the impact of intensive and local anthropisation.

Environmental DNA (eDNA) metabarcoding has emerged as a transformative approach for biodiversity assessment in such complex environments. By extracting DNA from environmental samples (e.g., water, sediment, etc.) and using high-throughput sequencing to match sequences against reference databases, it offers a non-invasive, sensitive, and taxonomically broad method for detecting species, including rare or elusive taxa [28–36]. In the Arabian Gulf, eDNA studies remain limited and largely taxon-specific, with applications including oil pollution assessment via benthic microbial communities [37,38] and the use of benthic foraminifera for environmental quality evaluation [1,39]. One study applied eDNA metabarcoding to survey marine vertebrates in Qatar [40], detecting a wide range of taxa, including cartilaginous fishes, bony fishes, turtles, birds, and mammals. Habitat type significantly influenced eDNA composition, with mangroves emerging as the most distinct habitat, and species compositions closely reflecting the known ecological preferences of each taxon. Efforts that support and facilitate such eDNA applications include the development of regional reference databases [24].

Beyond compiling taxonomic inventories, eDNA metabarcoding enables calculation of biodiversity metrics and indicators that link biodiversity science with environmental policy [41]. In UMEs, where selective pressures and habitat filtering are pronounced, combining taxonomic richness with phylogenetic diversity can reveal hidden aspects of community structure [42,43].

Here, we present the first eDNA-based survey of marine vertebrate biodiversity within the UME of Abu Dhabi. Using a standardized workflow of water sampling, eDNA metabarcoding, and taxonomic assignment [32], we surveyed vertebrate biodiversity in four chosen sites: (*i*) the Mangrove National Park, a protected intertidal ecosystem dominated by *Avicennia marina*, providing nursery habitat, organic matter input, and structural complexity known to support high vertebrate diversity [44], (*ii*) a seagrass bed near the city, representative of shallow subtidal seagrass beds that serve as feeding and resting areas for megafauna, such as dugongs and green sea turtles [45], as well as fish nursery site, (*iii*) the Marsa Al Bateen marina, a semi-enclosed, heavily modified coastal facility integrated into Abu Dhabi’s urban fabric, potentially characterized by reduced water flow, high pollution levels, and heavy maritime traffic—conditions known to harm native biodiversity, and (*iv*) the Al Hudayriyat channel, a recently developed coastal area whose infrastructure is still evolving; it thus offers a glimpse of a disturbed habitat at an early stage, where the community is likely still forming.

Given the ecological characteristics and levels of human disturbance at each site, and despite their geographical proximity, we expected vertebrate diversity and community composition to vary considerably among the four sites. The Mangrove National Park was expected to exhibit the highest alpha diversity and the most distinct assemblage, consistent with the role of mangroves as biodiversity hotspots. The seagrass bed was expected to harbor a functionally distinct assemblage, potentially allowing for the detection of megafauna and herbivorous fish species, despite the proximity of the Abu Dhabi UME. At the marina, reduced species richness was expected, with a potential increase in generalist and possibly non-native taxa reflecting the selective pressures of a heavily modified environment. The Al Hudayriyat channel, as a recently developed site, was expected to exhibit the lowest overall diversity, with a potentially disorganized community composition reflecting the habitat transition.

Based on the results of eDNA sampling and analysis, we conducted both taxonomic and phylogenetic beta diversity analyses to assess differences in community composition among sites. Our aim was to compute a set of diversity metrics, derived from eDNA metabarcoding data, that are relevant for biodiversity monitoring in a UME of the UAE. By doing so, this study addresses critical knowledge gaps on UAE marine biodiversity and provides a baseline for long-term, integrative biodiversity monitoring in the region.

## Materials and methods

### Study area and sampling design

**Fig 1:**
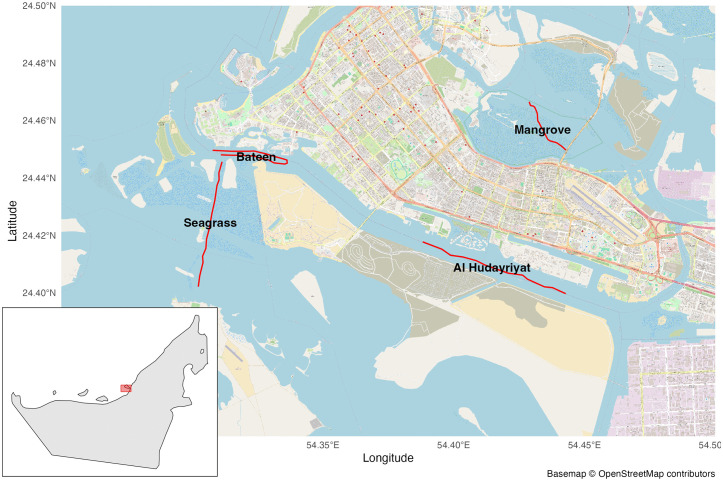
Map showing the location of the transects sampled around Abu Dhabi. Site names are indicated next to their respective transect (in red). Map created in R using maptiles and OpenStreetMap basemap data © OpenStreetMap contributors, https://www.openstreetmap.org/copyright.

Sampling took place during a dedicated campaign organised by the Sorbonne University Abu Dhabi (SUAD) Ocean Institute and the Environmental Agency - Abu Dhabi (EAD) on 16–17 December 2024. Four coastal stations were selected (Fig 1) and accessed using EAD’s coastal research vessel. The fieldwork was covered by Permit No. 200107179, issued by the Environment Agency of Abu Dhabi on October 8, 2024.

The Al Bateen station was located near Marsa Al Bateen Marina, a major marina bordered by extensive coastal urban developments. Al Hudayriyat station was situated within the Hudayriyat Channel, an area historically inhabited by Arabian pearl divers and recently transformed into an activity hub focused on outdoor sports and leisure. Mangrove station was located inside the Mangrove National Park, a key urban green space of 19 square kilometer of forest area that supports ecotourism activities such as kayaking and other environmentally friendly water sports. Seagrass station was situated in the Al Futaisi Channel area, adjacent to the northern part of Futaisi Island, a shallow, sheltered lagoonal complex southwest of Abu Dhabi Island. This privately-owned island, despite lacking freshwater sources, supports a rich diversity of habitats including sabkha (salt flats), intertidal flats, mangroves, and subtidal seagrass beds.

### Sample collection and laboratory processing

At each station, surface water samples were collected aboard a motorised rigid boat at a speed of ~5 knots using the following device: water was continuously filtered for 30 minutes using an Athena peristaltic pump (Proactive, Hamilton, NJ, USA) and a pipe. One end of the pipe was weighted with a 2-kg lead so that it would sink below the water’s surface, and the other end was connected to a 0.2 µm VigiDNA filtration capsules (SPYGEN, France) following a previously described protocol [32,33]. Water was filtered in duplicate (one filter per side of the boat). Immediately after filtration, capsules were filled with 80 mL of CL1 DNA preservation buffer (SPYGEN) and stored at room temperature until DNA extraction.

To avoid field contamination, strict precautions were taken during sampling. All tubes and filtration capsules were single-use and prepared in a “DNA-free” laboratory. The scientists wore single-use gloves, which were changed at least every time they opened a package of tubes or capsules. The work surface on the boat was covered with a large plastic sheet, which was cleaned with bleach immediately before each use.

DNA analysis was performed following the protocol described in [32,33] in a dedicated clean-rooms laboratory. Briefly, amplification targeted the mitochondrial 12S rRNA gene using the universal vertebrate primers Vert01 (Forward: TTAGATACCCCACTATGC; Reverse: TAGAACAGGCTCCTCTAG; [30]). Amplicons were sequenced on an Illumina MiSeq platform (Illumina, San Diego, CA, USA).

To monitor any potential laboratory contamination, negative controls were included during the DNA extraction and the PCR amplification and were sequenced in parallel with the samples.

Raw sequences were processed with the OBITools software suite [46], following the protocol described in [47]. Forward and reverse reads were first merged, then demultiplexed and dereplicated. Each PCR replicate per sample was segregated into a distinct dataset by partitioning the original dataset into multiple files. Sequences shorter than 20 bp, occurring fewer than 10 times per sample, or identified as “internal” by the obiclean program were excluded. Taxonomic assignment was performed using the ecotag program, against a reference database generated by *in silico* PCR with ecoPCR [48] on the GenBank nucleotide database (release 265), using the primer pair employed in this study. To avoid over-confident taxonomic assignments, we applied a hierarchical set of identity thresholds: species-level assignments were validated only for sequences with an identity match ≥ 98%, genus-level for 96–98%, family-level for 90–96%, and all sequences below 90% were discarded. To account for the potential misassignment of sequences to samples due to tag-jumps [49], we discarded all sequences with a frequency of occurrence < 0.001 per sequence and per library. We further corrected for index-hopping [50] using a threshold empirically determined for each sequencing batch from experimental blanks (i.e., tag combinations not present in the libraries).

Each sequence was then transformed to MOTUs (Molecular Operational Taxonomic Unit) with a unique identifier (AbuDhabi_C1_n°MOTU). To construct the biodiversity inventories, taxonomic assignments of each MOTU were meticulously checked by hand, considering molecular, taxonomic, and ecological criteria [51]. In most cases, one MOTU corresponded to a single distinct taxon, determined at the species level whenever possible. In a few cases of taxonomic redundancies, a single species was inferred from two or more MOTUs.

All resulting data were standardized and published on GBIF under a CC-BY 4.0 license: https://www.gbif.org/dataset/70a53441-4e44-4216-aac3-4e9d02e33212. Metadata followed the Darwin Core standard and MIMARKS guidelines [52–54]. Data were processed using the FAIRe suite [55], including FAIRe-ator, FAIRe-fier, and FAIRe2MDT, and published through the GBIF Metabarcoding Data Toolkit [56].

### Taxonomic and phylogenetic diversity analyses

The lists of taxa detected at each sampling station were transformed into a binary presence–absence matrix. All statistical analyses were performed using R version 4.4.3 [57], and the code used to reproduce all analyses and figures is available on GitHub: https://github.com/HaderleRachel/eDNA_AbuDhabi_Dec2024/tree/main.

#### Taxonomic and phylogenetic β-diversity.

Taxonomic β-diversity was quantified using Jaccard dissimilarity (*D*_*taxo*_ = 0 for identical sites, *D*_*taxo*_ = 1 for sites sharing no taxa) and decomposed into turnover and nestedness components using the *beta.div.comp* function from the *adespatial* package [58,59]. Turnover reflects species replacement between communities, while nestedness captures differences in species richness due to species loss or gain.

A comprehensive vertebrate phylogenetic megatree was constructed using the *U.PhyloMaker* package [60]. The megatree combined an actinopterygian phylogeny [61], a chondrichthyan phylogeny [62], a bird phylogeny [63], and a mammalian phylogeny [64]. Phylogenetic β-diversity was assessed using the UniFrac index, which integrates phylogenetic distances [65], computed using the *unifrac* function from the *picante* package [66]. UniFrac also allowed us to decompose the distances into turnover (phylogenetic replacement) and nestedness (loss of phylogenetic branches).

Principal Component Analyses (PCA) were performed on β-diversity components using the *factoextra* package [67] to identify patterns of similarity across sites.

#### Phylogenetic diversity metrics.

Three complementary facets of phylogenetic diversity were calculated. Faith’s Phylogenetic Diversity (PD), defined as the total branch length connecting all taxa detected at a site [68], and computed using the *picante* package [69]. Mean Pairwise Distance (MPD), which measures the average phylogenetic distance among all pairs of taxa [70], thereby capturing deeper evolutionary divergences [71]. Variance in Pairwise Distance (VPD), measuring the variance in phylogenetic distances among species [72,73], capturing more complex phylogenetic structure such as the presence of distinct old lineages with recent clusters of closely related taxa [73,74].

A standardized effect size (SES) was calculated for the three indices using the *picante* package [69] by comparing observed values to a null distribution (n = 1,000 random trees), generated by shuffling species labels at the tree tips [75]. Values significantly different from the null hypothesis were determined using the 95% percentile interval of a normalized Gaussian distribution.

## Results

### Taxonomic assignment and biodiversity inventory

A total of 83 distinct taxa (94 MOTUs) were identified, including five bird species, two elasmobranchs, one marine mammal, and 75 teleost taxa (Table 1). Twenty-two taxa were assigned to the species level.

**Table 1:** List of taxa detected in each sample.

Class	Family	Identified taxon	Number of possible species (see A1)	IUCN status, commercial importance (for teleosts) and any naturalist comments	Al_Hudayriyat	Bateen	Mangrove	Seagrass
Aves	Laridae	Laridae			0	0	1	1
Aves	Laridae	*Larus* sp.			0	0	0	1
Aves	Ardeidae	*Butorides striata*		Least Concern	0	0	1	0
Aves	Columbidae	Columbidae			0	0	1	0
Aves	Phalacrocoracidae	*Phalacrocorax* sp.			0	0	1	0
Elasmobranchii		Carcharhiniformes	8		1	0	0	0
Elasmobranchii	Dasyatidae	*Pastinachus sephen*		Near Threatened, minor commercial	0	1	0	0
Mammalia	Delphinidae	Delphinidae	7		1	1	0	1
Teleostei	Ephippidae	*Platax* sp.	2	Least Concern, minor commercial	1	1	0	1
Teleostei	Leiognathidae	Leiognathidae	2	Data Deficient, minor commercial	1	1	0	1
Teleostei	Pomacanthidae	*Pomacanthus maculosus*		Least Concern, commercial	1	1	1	1
Teleostei	Siganidae	*Siganus* sp.	3	Least Concern	1	1	1	1
Teleostei	Atherinidae	*Atherinomorus lacunosus*		Least Concern, commercial	1	1	1	1
Teleostei	Belonidae	*Tylosurus crocodilus*		Least Concern, commercial	1	1	1	1
Teleostei	Hemiramphidae	*Hemiramphus* sp*._1*	2	Commercial	0	0	1	0
Teleostei	Hemiramphidae	*Hemiramphus* sp*._2*	2	Commercial	1	1	1	1
Teleostei	Blenniidae	Blenniidae	2		1	1	0	1
Teleostei	Sphyraenidae	*Sphyraena* sp*._1*	2	Commercial	1	0	1	0
Teleostei	Sphyraenidae	*Sphyraena* sp*._2*	2	Commercial	1	0	1	0
Teleostei	Sphyraenidae	*Sphyraena* sp*._3*	2	Commercial	1	1	1	1
Teleostei	Carangidae	Carangidae	6	Least Concern	1	1	1	1
Teleostei	Carangidae	*Alepes* sp*._1*	3	Least Concern	1	0	0	0
Teleostei	Carangidae	*Alepes* sp*._2*	3	Least Concern	1	0	0	0
Teleostei	Carangidae	*Gnathanodon speciosus*		Least Concern, minor commercial, priority species (Wabnitz et al., 2018)	1	1	1	1
Teleostei	Carangidae	*Scomberoides* sp.	3	Least Concern, minor commercial	1	1	1	1
Teleostei	Terapontidae	*Pelates quadrilineatus*		Minor commercial, priority species (Wabnitz et al., 2018)	1	1	0	0
Teleostei	Terapontidae	*Terapon puta*		Minor commercial	1	1	1	1
Teleostei	Cichlidae	*Oreochromis* sp*._1*	1	Least Concern, commercial, MOTUs AbuDhabi_C1_26 and AbuDhabi_C1_249	1	1	0	0
Teleostei	Cichlidae	*Oreochromis* sp*._2*	3		1	0	0	0
Teleostei		Clupeiformes_1	4		1	1	1	1
Teleostei		Clupeiformes_2	3		1	0	0	1
Teleostei	Dorosomatidae	*Nematalosa* sp.	2	Minor commercial	1	1	0	1
Teleostei	Clupeidae	*Sardinella* sp*._1*	2	Least Concern	0	0	1	0
Teleostei	Clupeidae	*Sardinella* sp*._2*	3	Least Concern	0	0	1	0
Teleostei	Engraulidae	*Encrasicholina heteroloba*		Least Concern, minor commercial	0	1	1	0
Teleostei	Cyprinodontidae	*Aphaniops stoliczkanus*		MOTUs AbuDhabi_C1_240 and AbuDhabi_C1_255	0	0	1	0
Teleostei	Gerreidae	*Gerres* sp*._1*	2	Commercial	1	1	1	1
Teleostei	Gerreidae	*Gerres* sp*._2*	2	Commercial	0	1	0	1
Teleostei	Gerreidae	*Gerres* sp*._3*	2	Commercial	1	1	1	1
Teleostei	Haemulidae	*Plectorhinchus* sp*._1*	1	Least Concern, commercial	1	0	0	0
Teleostei	Haemulidae	*Plectorhinchus* sp*._2*	2	Least Concern, commercial	1	1	1	1
Teleostei	Haemulidae	*Pomadasys* sp.	5	Least Concern, commercial	1	0	1	0
Teleostei	Lethrinidae	*Lethrinus* sp.	3	Least Concern	1	1	1	1
Teleostei	Lutjanidae	*Lutjanus* sp*._1*	2	Least Concern, commercial	0	0	1	0
Teleostei	Lutjanidae	*Lutjanus* sp*._2*	3	Least Concern,	1	1	1	1
Teleostei	Lutjanidae	*Lutjanus* sp*._3*	3	Least Concern, commercial	1	1	1	1
Teleostei	Monodactylidae	*Monodactylus argenteus*		Least Concern, minor commercial	0	1	0	1
Teleostei	Nemipteridae	Nemipteridae_1	4	Least Concern	1	1	0	1
Teleostei	Nemipteridae	Nemipteridae_2	4	Least Concern	1	1	1	1
Teleostei	Sillaginidae	*Sillago sihama*		Least Concern, commercial, MOTUs AbuDhabi_C1_186 and AbuDhabi_C1_187	1	1	1	0
Teleostei	Sparidae	*Acanthopagrus* sp*._1*	2	Least Concern, MOTUs AbuDhabi_C1_136, AbuDhabi_C1_137 and AbuDhabi_C1_140	1	1	1	1
Teleostei	Sparidae	*Acanthopagrus* sp*._2*	1	Least Concern, commercial, MOTUs AbuDhabi_C1_138 and AbuDhabi_C1_139	1	1	1	1
Teleostei	Sparidae	*Diplodus kotschyi*		Minor commercial	1	0	0	0
Teleostei	Sparidae	*Pagellus* sp.	1	Least Concern, commercial	1	1	1	1
Teleostei	Sparidae	*Rhabdosargus* sp.	2	Least Concern, commercial	1	1	1	1
Teleostei		Gobiiformes_1	19	Least Concern	1	1	0	1
Teleostei		Gobiiformes_2	19	Least Concern	0	0	0	1
Teleostei	Gobiidae	Gobiidae_1	9	Least Concern, commercial	0	0	0	1
Teleostei	Gobiidae	Gobiidae_2	9	Least Concern, commercial	0	0	1	0
Teleostei	Gobiidae	Gobiidae_3	9	Least Concern, commercial	1	1	1	1
Teleostei	Gobiidae	Gobiidae_4	2	Least Concern, commercial	0	0	0	1
Teleostei	Gobiidae	*Cryptocentrus lutheri*		Least Concern	1	0	1	1
Teleostei	Gobiidae	*Favonigobius melanobranchus*		Near Threatened	1	1	1	1
Teleostei	Gobiidae	*Valenciennea* sp.	1	Least Concern, commercial, MOTUs AbuDhabi_C1_81 and AbuDhabi_C1_82	1	1	1	1
Teleostei	Chanidae	*Chanos chanos*		Least Concern, highly commercial, priority species (Wabnitz et al., 2018)	1	0	1	1
Teleostei	Apogonidae	Apogonidae	2		1	0	0	0
Teleostei	Apogonidae	*Apogonichthyoides* sp.	1	MOTUs AbuDhabi_C1_92, AbuDhabi_C1_94 and AbuDhabi_C1_95	1	0	1	1
Teleostei	Mugilidae	Mugilidae	2	Data Deficient, commercial	0	1	0	0
Teleostei	Mugilidae	*Moolgarda seheli*			0	0	1	0
Teleostei	Mullidae	*Parupeneus* sp.	3	Least Concern, commercial	0	1	1	1
Teleostei	Mullidae	*Upeneus* sp*._1*	3	Commercial	1	1	1	1
Teleostei	Mullidae	*Upeneus* sp*._2*	3	Commercial	1	0	0	1
Teleostei	Pseudochromidae	*Pseudochromis* sp.	4	Least Concern, commercial	1	0	1	1
Teleostei		Perciformes	3	Least Concern, minor commercial	0	0	1	0
Teleostei	Epinephelidae	*Epinephelus* sp.	2	Least Concern, commercial	1	1	1	1
Teleostei	Platycephalidae	*Platycephalus indicus*		Data Deficient, commercial, MOTUs AbuDhabi_C1_114 and AbuDhabi_C1_115, priority species (Wabnitz et al., 2018)	1	1	1	1
Teleostei	Paralichthyidae	Paralichthyidae	2	Least Concern, commercial	0	1	0	0
Teleostei	Soleidae	Soleidae	2	Commercial	1	0	0	1
Teleostei	Scombridae	*Rastrelliger kanagurta*		Data Deficient, highly commercial, priority species (Wabnitz et al., 2018)	1	0	0	0
Teleostei	Scombridae	*Scomberomorus commerson*		Near Threatened, highly commercial, AbuDhabi_C1_121 and AbuDhabi_C1_122, priority species (Wabnitz et al., 2018)	1	1	1	1
Teleostei	Plotosidae	*Plotosus lineatus*		Commercial	0	1	0	0
Teleostei	Monacanthidae	Monacanthidae	4	Least Concern, minor commercial	1	1	1	1
Teleostei	Triacanthidae	*Triacanthus biaculeatus*		Minor commercial	1	1	1	1

0: taxon not detected, 1 (and gray color): taxon detected in this place.

Notable identifications include the cowtail stingray *Pastinachus sephen* (Al Bateen), which is classified as Near Threatened on the IUCN Red List. Several priority species [6] were detected: the golden kingfish *Gnathanodon speciosus* (all sites), the fourlined terapon *Pelates quadrilineatus* (Al Hudayriyat and Al Bateen), the milkfish *Chanos chanos* (highly commercial; all sites except Al Bateen), the bartail flathead *Platycephalus indicus* (all sites), the Indian mackerel *Rastrelliger kanagurta* (highly commercial; only Al Hudayriyat), and the narrow-barred Spanish mackerel *Scomberomorus commerson* (highly commercial, Near Threatened and priority species; all sites). Among them are several pelagic species of commercial importance, including the Indian and the Spanish mackerel**.** The Arabian killifish (*Aphaniops stoliczkanus*), a freshwater species tolerant to salinity, has been detected in the mangrove.

Five bird taxa were detected: three taxa not identifiable to the species level (two belonging to the Laridae family, and one to the Columbidae), one *Phalacrocorax sp.*, most likely *P. nigrogularis*, the Socotra cormorant, which is globally listed as Vulnerable on the IUCN Red List, and the green-backed heron, *Butorides striata*, also observed during sampling in the mangrove habitat.

**Fig 2:**
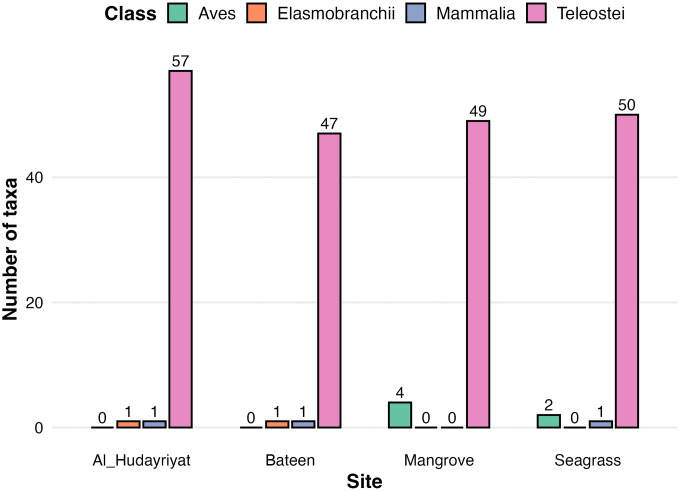
Number of taxa in each class detected for each station.

Site-specific taxonomic richness ranged from 49 taxa at Al Bateen to 59 at Al Hudayriyat, with both Mangrove and Seagrass each yielding 53 taxa (Fig 2). Birds were not detected in Al Bateen and Al Hudayriyat, but four of the five species were recorded in the mangrove and two in seagrass. Elasmobranchs included one *Carcharhiniformes* (likely *Carcharhinus* sp. or *Sphyrna* sp.) at Al Hudayriyat and *Pastinachus sephen* at Al Bateen. Marine mammals were represented by Delphinidae, most likely *Sousa plumbea*, detected at all sites except the mangrove (Fig 2), although confirmation was not possible due to the absence of a complete reference barcode in GenBank.

**Fig 3:**
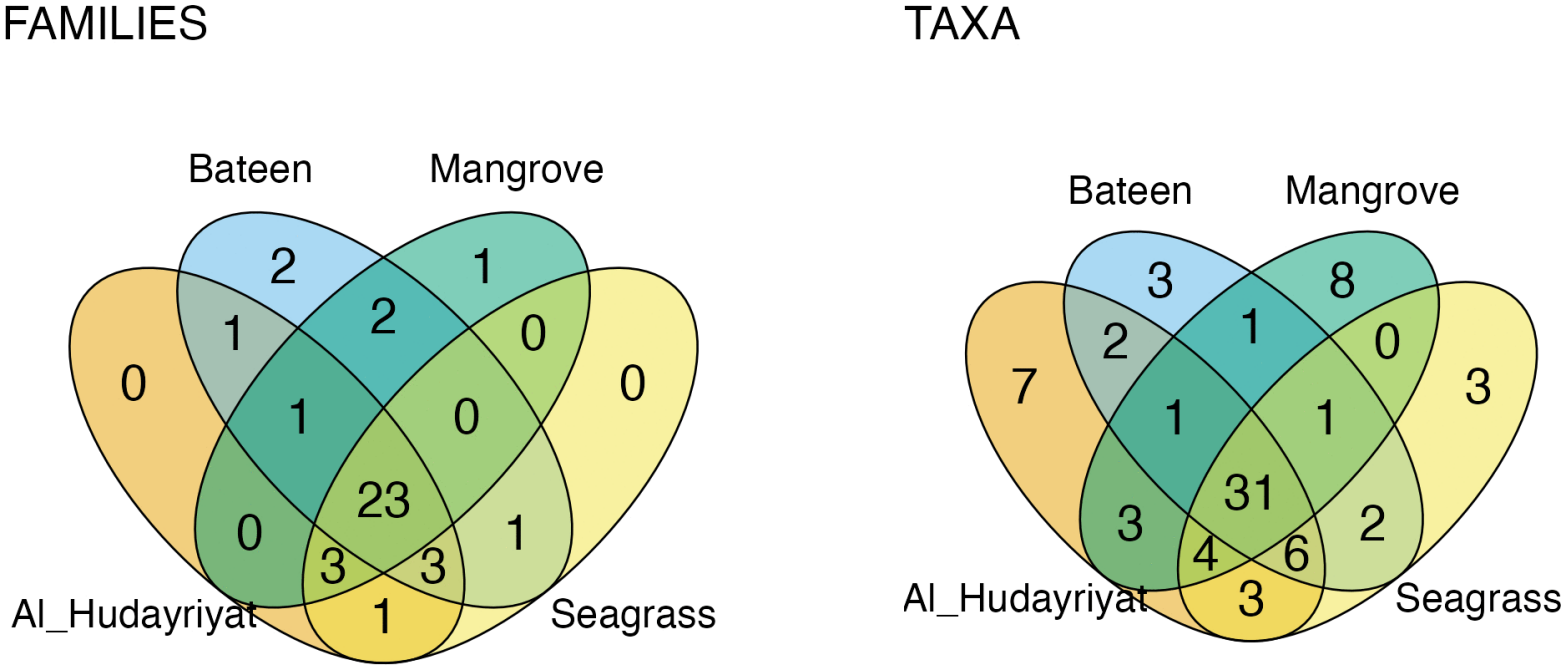
Venn diagrams showing the number of shared and unique teleost families (left) and assigned taxa (right) detected across four sampling sites.

When focusing exclusively on teleosts, 23 families were shared among the four sites (Fig 3, S1 Appendix), representing a large proportion of the overall assemblages. Although the proportions of shared families were relatively similar across sites, the highest overlap was observed between Al Hudayriyat and Al Bateen, with 28 families in common. A small number of families were unique to specific sites: two at Al Bateen (Paralichthyidae and Plotosidae) and one at Mangrove (Cyprinodontidae).

A similar pattern was observed at the taxonomic level (Fig 3): 31 teleost taxa were common to all four sites, with the maximum overlap again between Al Hudayriyat and Al Bateen (40 taxa shared). However, several taxa were unique to single sites, including eight at Mangrove, seven at Al Hudayriyat, and three each at Al Bateen and Seagrass.

Across sites, reef-associated teleosts were the most abundant group, accounting for ~60% of the species detected. Demersal fish represented the second most frequent group, ranging from 18% of taxa at Mangrove to 24% at Al Bateen.

### Differences between sites and beta diversity indices

Taxonomic dissimilarity was generally moderate to low across sites (Table 2). The maximum dissimilarity (*D*_*taxo*_ = 0.50) was observed between Al Bateen and Mangrove, whereas the lowest (*D*_*taxo*_ = 0.33) occurred between Seagrass and Al Hudayriyat, and between Seagrass and Al Bateen. Overall, the mangrove site showed the greatest dissimilarity with the other habitats, while Al Hudayriyat and Al Bateen exhibited the highest pairwise dissimilarity, mangrove excluded (*D*_*taxo*_ = 0.39).

**Table 2:** Pairwise dissimilarity matrices for taxonomic β-diversity between sites.

	Al Hudayriyat	Al Bateen	Mangrove
Al Bateen	0.39		
Mangrove	0.47	0.50	
Seagrass	0.33	0.33	0.46

Partitioning of β-diversity indicated that turnover was the dominant component (82%), while nestedness accounted for 18% of overall dissimilarity (S2 Appendix).

Phylogenetic dissimilarity values were broadly consistent with taxonomic results (Table 3). The highest dissimilarity (*D*_*phylo*_ = 0.55) was again between Al Bateen and Mangrove, and the lowest (*D*_*phylo*_ = 0.27) between Seagrass and Al Hudayriyat. As with taxonomic dissimilarity, the mangrove site showed the greatest dissimilarity relative to the other sites.

**Table 3:** Pairwise dissimilarity matrices for phylogenetic β-diversity between sites.

	Al Hudayriyat	Al Bateen	Mangrove
Bateen	0.30		
Mangrove	0.46	0.55	
Seagrass	0.27	0.42	0.43

Turnover largely explained 87% of overall phylogenetic dissimilarity, whereas nestedness contributed only 13%, and turnover was dominant in all pairwise comparisons (S2 Appendix).

**Fig 4:**
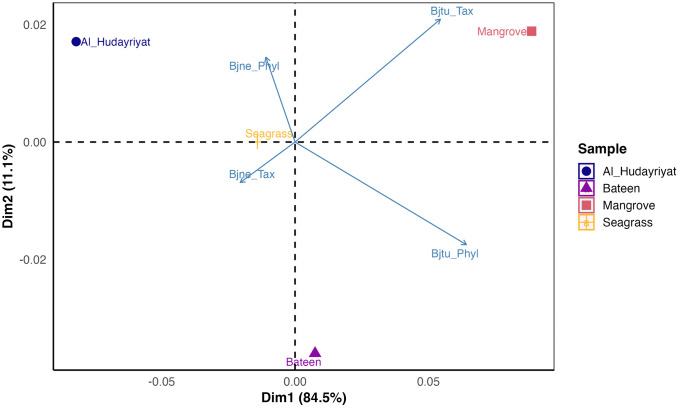
PCA based on taxonomic and phylogenetic β-diversity (turnover and nestedness components).

The PCA revealed a clear structuring of sites along the first axis, which explained 84.5% of the total variance (Fig 4). Mangrove and Al Hudayriyat were the most distinct sites along the horizontal axis, with the separation of Mangrove primarily driven by taxonomic turnover. In contrast, Seagrass and Al Bateen clustered more closely together on this horizontal axis, with Al Bateen differing from the other sites on the vertical axis.

### Phylogenetic diversity metrics

Phylogenetic diversity (PD) reached its maximum at Mangrove (*PD*_*Mangrove*_ = 3931), followed by Seagrass (*PD*_*Seagrass*_ = 3639), Al Bateen (*PD*_*Bateen*_ = 3608), and was lowest at Al Hudayriyat (*PD*_*Al_Hudayriyat*_ = 3223). Mean pairwise distance (MPD) was highest at Mangrove (*MPD*_*Mangrove*_ = 267) and lowest at Al Hudayriyat (*MPD*_*Al_Hudayriyat*_ = 195), with intermediate values at Seagrass (*MPD*_*Seagrass*_ = 242) and Bateen (*MPD*_*Bateen*_ = 231). Variance in phylogenetic diversity (VPD) was greatest at Seagrass (*VPD*_*Seagrass*_ = 42,503) and Mangrove (*VPD*_*Mangrove*_ = 42,145), but markedly lower at Bateen (*VPD*_*Bateen*_ = 32,082) and Al Hudayriyat (*VPD*_*Al_Hudayriyat*_ = 16,101).

**Fig 5:**
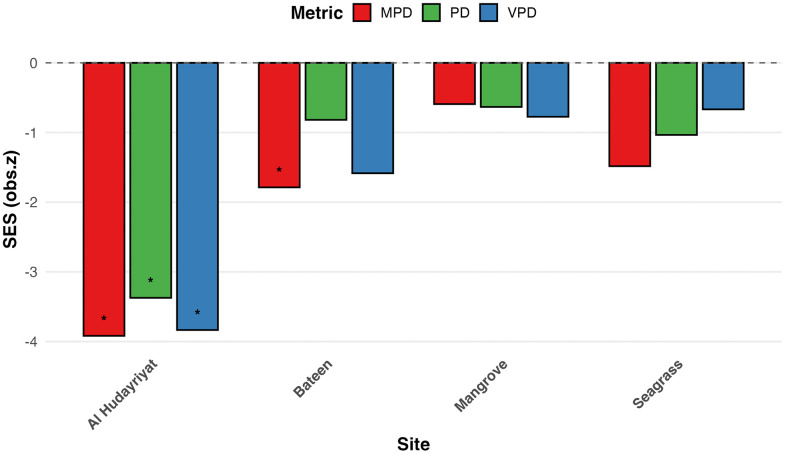
Standardized effect size (SES) values for mean pairwise distance (MPD), phylogenetic diversity (PD), and variance in phylogenetic diversity (VPD) across sites. Asterisks denote significant deviations (p < 0.05).

Standardized effect size (SES) metrics (Fig 5) generally reflected these patterns but also revealed whether observed values deviated from expectations given taxonomic richness. Al Hudayriyat showed significantly lower values for all indices (*p* = 0.001 for the three indices). At Al Bateen, SES.MPD (*p* = 0.046) was also significantly lower than expected, whereas SES.VPD was the second-lowest value among sites but not significant (*p* = 0.062). By contrast, SES values at Mangrove, and to a lesser extent at Seagrass, did not differ significantly from expectations, suggesting that their observed phylogenetic structure was consistent with random assemblage patterns given species richness.

## Discussion

This study represents the first eDNA-based assessment of marine vertebrate biodiversity within the urban marine ecosystem (UME) of Abu Dhabi. Our primary objectives were to (*i*) provide a comprehensive inventory of vertebrate taxa across contrasting urban coastal habitats (including one mangrove and one seagrass), (*ii*) quantify both taxonomic and phylogenetic diversity to better understand community structure; and (*iii*) combine standard taxonomic metrics with complementary phylogenetic measures, Faith’s Phylogenetic Diversity (PD), Mean Pairwise Distance (MPD), and Variance in Pairwise Distance (VPD), along with their standardized effect sizes (SES) to capture multiple dimensions of biodiversity. In addition, this assessment is intended to serve as a basis for future comparison with periods of heat stress in summer and for analyzing interannual variations.

### Revealing Abu Dhabi’s coastal biodiversity through eDNA

Our survey provides the first eDNA-based taxonomic survey of vertebrate biodiversity in Abu Dhabi’s coastal ecosystems. In total, we identified 75 distinct teleost taxa, representing approximately 15% of the 485 teleost species reported from the UAE [76], and more than three times the number of species reported locally [77]. Only about one-quarter of detections could be resolved to the species level, reflecting current limitations in reference databases. Indeed, only 52% of Arabian Gulf marine fishes are currently represented for the selected barcode in public repositories (https://shiny.cefe.cnrs.fr/GAPeDNA/ [78]). Valuable initiatives have recently strongly enriched the development of fish reference libraries, such as the COI barcode dataset previously compiled in 2020 from 164 trawl and trap stations in United Arab Emirates waters [24]. But significant gaps remain and continue to limit species attributions. These gaps can be filled in the coming years. But although sequencing costs have decreased substantially, the development of such a database still requires long-term specimen collection efforts and taxonomic identification based on the involvement and expertise of local specialists for the relevant taxonomic groups. Building a local curated reference database focused particularly on marine fishes, is one of our planned initiatives for the coming years.

Despite these limitations, our dataset recovered more than 40% of the families reported previously in the UAE [24] and yielded 13 perfect matches (representing 65% of the 20 teleosts identified to the species level through eDNA), a satisfactory outcome given our more restricted sampling coverage and nearshore focus. Additionally, eight families were detected exclusively through eDNA, highlighting the complementary value of molecular surveys for characterizing coastal fish biodiversity. Cryptobenthic fish, which are small, difficult to observe, and may be overlooked by other study methods, are a good example of the added value of eDNA approaches. The relatively low percentage of MOTUs assigned to the species level in our results should nevertheless have a relatively limited impact on the conclusions. Future improvements in taxonomic assignments would not significantly alter the trends observed, for example, in taxonomic β-diversity, particularly because the MOTUs shared across sites would remain the same. Phylogenetic diversity calculation could become more precise, but with no possibility of calling the patterns into question.

Beyond teleosts, eDNA metabarcoding enabled the detection of one cetacean, most likely the Indian Ocean humpback dolphin (*Sousa plumbea*), identified at all sites except the mangrove. Confirmation was limited by incomplete reference barcodes in GenBank; however, a partial sequence (GB accession number OR545836) matched with 100% identity, albeit only 41% query coverage. This tentative assignment is reinforced by field observations of a pod near Al Bateen during sampling, as well as previous reports of frequent occurrence along Abu Dhabi’s coast [79]. The species’ nearshore distribution renders it highly vulnerable to anthropogenic threats such as maritime traffic and gill-netting [80,81]. Globally, *Sousa plumbea* is listed as Endangered by the IUCN [82], underscoring the conservation significance of this detection.

Elasmobranch diversity was comparatively low, with only two taxa identified: a Carcharhiniformes (likely *Carcharhinus sp.* or *Sphyrna sp.*) at Al Hudayriyat, and the cowtail ray (*Pastinachus sephen*) at Al Bateen. *Pastinachus sephen*, a benthic species of soft-substrate habitats, is common across UAE waters [83] and listed as Near Threatened due to bycatch pressures elsewhere in its range [84]. Limited taxonomic resolution for Carcharhiniformes may be overcome by multi-marker approaches [47,85] and by completing the reference databases.

We also detected several “priority species” for Gulf fisheries [6], particularly at Al Hudayriyat. This suggests that, despite extensive coastal development, Abu Dhabi waters remain important for both biodiversity and fisheries. Some pelagic detections at Al Bateen may reflect fishing discards, as recreational and artisanal fishing activities can bias eDNA signals that rely on trace DNA.

Taxonomic richness was relatively consistent across sites, with a mean of ~54 taxa per site. This represents a relatively high level of diversity, given the Gulf’s naturally low species richness compared to adjacent regions [21,86]. Such richness may also be explained by the close juxtaposition of key habitats. Mangroves, algae, and seagrass beds provide crucial refuge and forage opportunities for numerous marine species in the Gulf [87]. In particular, mangroves deliver essential ecological and physical services, functioning as nurseries for commercially important species [77,88,89] and serving as feeding and breeding grounds for a wide array of marine and terrestrial fauna.

### Fine-scale community shifts: What eDNA reveals about urban marine ecosystems

The four sites we sampled are close to each other, at most a few kilometers apart. Some are very close, such as Al Bateen and Al Hudayriyat, or Al Bateen and Seagrass (Fig 1). Nevertheless, while overall taxonomic richness was broadly similar, community composition revealed habitat-specific differences. The mangrove site emerged as the most distinct, home to eight unique taxa, including the Arabian killifish (*Aphaniops stoliczkanus*), a euryhaline species adapted to coastal lagoons [77]. This finding is consistent with a previous study, which reported mangroves as the most distinct habitat type in Qatar [40], likely reflecting their extreme environmental conditions of elevated temperature and salinity [90]. At Al Bateen, unique families included Paralichthyidae (flatfishes) and Plotosidae, the latter represented by *Plotosus lineatus*, the only coral reef catfish known in the region [76].

Beta-diversity analyses confirmed these compositional patterns, indicating that community differences among sites were primarily driven by turnover (species replacement) rather than nestedness (species loss). The Mangrove, the most geographically isolated site, exhibited the highest taxonomic and phylogenetic turnover. A strong similarity (second-lowest pairwise dissimilarity values) occurred between Al Bateen and Al Hudayriyat, despite their contrasting habitat types (a marina vs. a newly developed island-adjacent site). This may reflect anthropogenic homogenization of teleost communities in heavily developed coastal zones [91]. Seagrass and Al Bateen, although geographically close (<5 km), remained relatively distinct.

The detection of dissimilarities over short spatial scales demonstrates that eDNA is not evenly distributed, even at fine resolutions. It should be noted that a Mantel test using site coordinates revealed no significant relationship between distance of the sampling sites and taxonomic or phylogenetic dissimilarities. Comparable small-scale variations have been widely documented across aquatic ecosystems, including Swiss river networks [92], estuaries in western France [93], the Gulf of St. Lawrence [94], and coastal protected areas in the Moray Firth (Scotland) and the Iroise Sea (France) [36,95]. Our results add to this body of evidence by extending it to UME in the UAE. UMEs remain poorly understood, with largely undocumented biodiversity, particularly for vertebrates, and the mechanisms governing species assembly and turnover appear especially complex [96]. Coastal urbanization, through stressors such as chemical and noise pollution, habitat alteration, overexploitation, and the proliferation of artificial structures, disrupts natural habitats, reduces foundation species, favours opportunistic or non-native taxa, and imposes selective pressures that promote phenotypic plasticity and local adaptation [17,27].

### Phylogenetic diversity patterns distinguish mangroves and seagrass beds as biodiversity reservoirs in coastal areas of Abu Dhabi

Phylogenetic diversity provides complementary insights into the evolutionary history preserved within communities [97], with implications for ecosystem functioning and resilience [98,99].

Across sites, all phylogenetic metrics (and their standardized effect sizes) were consistently higher for the Mangrove and Seagrass sites, while Al Bateen and Al Hudayriyat showed lower values. Standardized effect sizes revealed lower-than-expected values for PD, MPD and VPD at Al Hudayriyat, indicating phylogenetic clustering and reduced divergence. At Al Bateen, the same pattern existed, but was less marked, with only MPD showing significantly lower-than-expected values. MPD reflects average evolutionary divergence among species, driven by ancient diversification events [71,100], while VPD captures variability in divergence, reflecting both recent and ancient splits [72]. Such clustering is typically indicative of environmental filtering, favoring closely related, disturbance-tolerant taxa [101]. This may be particularly relevant in the Gulf, where extreme environmental conditions (high temperature, salinity, and turbidity driven by arid geography and shallow bathymetry; [102]) already exert, together with pronounced anthropisation, strong selective pressures.

Phylogenetic clustering in anthropized sites suggests reduced niche complementarity and potentially compromised ecosystem functioning. This is consistent with evidence that phylogenetic diversity tends to decline with human disturbance [103]. Nonetheless, Al Bateen maintained non-negligible PD and VPD (as SES.PD and SES.VPD were not significantly lower), suggesting that artificial substrates in the marina can sustain some degree of evolutionary breadth. Indeed, ports and marinas may harbor unexpectedly high biodiversity due to the structural heterogeneity of artificial substrates, which create microhabitats and potential refugia [104,105]. Such environments may even serve as opportunistic refuges during extreme climatic events [42,106,107].

Conversely, mangrove and seagrass sites maintained relatively higher phylogenetic diversity. This likely reflects both the intrinsic ecological properties of these habitats and, for the mangrove site, its location within a protected national park. Similar patterns have been observed in Mediterranean eDNA surveys, where higher phylogenetic diversity was detected inside reserves [32]. These findings reinforce the role of mangroves and seagrass beds as biodiversity reservoirs, even under strong urban and anthropogenic pressures.

### Implications for biodiversity monitoring and urban marine management

UAE reefs and coastal habitats are exposed to multiple, interacting pressures, including rapid urban and industrial expansion, dredging, oil and gas activities, and accelerating climate change [17,108]. Regional marine biota already persists near their upper thermal and salinity tolerance limits [21,86], making biodiversity monitoring particularly urgent in this uniquely constrained UME. In this context, the Gulf serves as an exceptional natural laboratory to investigate the ecological consequences of global change within a semi-enclosed, environmentally extreme marine system.

Here, we provide the first eDNA-based inventory of vertebrate biodiversity across Abu Dhabi’s nearshore waters, spanning mangroves, seagrass beds, marinas, and artificial coastlines. Despite taxonomic limitations associated with incomplete reference libraries, we detected substantial richness, including threatened, commercially important, and ecologically significant species. Beta-diversity analyses revealed that community differences were driven predominantly by species turnover, indicating true replacement of taxa among habitats. Mangrove and seagrass sites supported distinct assemblages and consistently higher phylogenetic diversity, underlining their disproportionate contribution to the region’s evolutionary heritage.

Conversely, the phylogenetic clustering observed at Al Hudayriyat and, to a lesser extent, Al Bateen suggests the influence of strong environmental filtering in highly modified or recently constructed coastal habitats. Such patterns are consistent with the effects of urbanization, habitat alteration, and the extreme environmental conditions characteristic of the Gulf, which may collectively favor disturbance-tolerant and closely related lineages. Continuing this study over time and expanding it spatially are among the objectives of this work, as this will help clarify the temporal stability of these patterns and improve understanding of biodiversity responses to ongoing environmental change.

Collectively, these findings establish a critical baseline for assessing biodiversity change in the region and highlight the value of combining eDNA-based taxonomic inventories with phylogenetic metrics to gain an integrated understanding of community structure [35]. This approach provides a powerful, non-invasive framework for long-term monitoring, supports evidence-based conservation planning, and will be essential for managing and safeguarding coastal vertebrate communities in the UAE under the broader context of global change.

## Supporting information

S1 Appendix List of teleost families and taxa shared among and unique to the sites represented in the Venn diagram (Fig 3). (PDF)

S2 Appendix Matrix showing turnover (above the diagonal) and nestedness (below the diagonal) for taxonomic and phylogenetic beta-diversity. (PDF)
