# Supplementary material for: First eDNA-based monitoring of marine vertebrates in the urban marine ecosystem of Abu Dhabi: Mangrove and seagrass beds as biodiversity reservoirs?
Source: PLoS One. 2026 Aug 4;21(8):e0352249. doi: 10.1371/journal.pone.0352249 (PMC13436779; doi:10.1371/journal.pone.0352249)
Supplement: Supplementary file 1 [file pone.0352249.s001.pdf]

Appendix 1: List of teleost families and taxa shared among and unique to the sites represented in the Venn diagram (Figure 3)

FAMILY	Sites	N_sites
Sparidae	Al_Hudayriyat,Bateen,Mangrove,Seagrass	4
Carangidae	Al_Hudayriyat,Bateen,Mangrove,Seagrass	4
Atherinidae	Al_Hudayriyat,Bateen,Mangrove,Seagrass	4
	Al_Hudayriyat,Bateen,Mangrove,Seagrass	4
Gobiidae	Al_Hudayriyat,Bateen,Mangrove,Seagrass	4
Epinephelidae	Al_Hudayriyat,Bateen,Mangrove,Seagrass	4
Gerreidae	Al_Hudayriyat,Bateen,Mangrove,Seagrass	4
Hemiramphidae	Al_Hudayriyat,Bateen,Mangrove,Seagrass	4
Lethrinidae	Al_Hudayriyat,Bateen,Mangrove,Seagrass	4
Lutjanidae	Al_Hudayriyat,Bateen,Mangrove,Seagrass	4
Monacanthidae	Al_Hudayriyat,Bateen,Mangrove,Seagrass	4
Dorosomatidae	Al_Hudayriyat,Bateen,Mangrove,Seagrass	4
Nemipteridae	Al_Hudayriyat,Bateen,Mangrove,Seagrass	4
Terapontidae	Al_Hudayriyat,Bateen,Mangrove,Seagrass	4
Platycephalidae	Al_Hudayriyat,Bateen,Mangrove,Seagrass	4
Haemulidae	Al_Hudayriyat,Bateen,Mangrove,Seagrass	4
Pomacanthidae	Al_Hudayriyat,Bateen,Mangrove,Seagrass	4
Scombridae	Al_Hudayriyat,Bateen,Mangrove,Seagrass	4
Siganidae	Al_Hudayriyat,Bateen,Mangrove,Seagrass	4
Sphyraenidae	Al_Hudayriyat,Bateen,Mangrove,Seagrass	4
Triacanthidae	Al_Hudayriyat,Bateen,Mangrove,Seagrass	4
Belonidae	Al_Hudayriyat,Bateen,Mangrove,Seagrass	4
Mullidae	Al_Hudayriyat,Bateen,Mangrove,Seagrass	4
Apogonidae	Al_Hudayriyat,Mangrove,Seagrass	3
Blenniidae	Al_Hudayriyat,Bateen,Seagrass	3
Chanidae	Al_Hudayriyat,Mangrove,Seagrass	3
Leiognathidae	Al_Hudayriyat,Bateen,Seagrass	3
Ephippidae	Al_Hudayriyat,Bateen,Seagrass	3
Pseudochromidae	Al_Hudayriyat,Mangrove,Seagrass	3
Sillaginidae	Al_Hudayriyat,Bateen,Mangrove	3
Cichlidae	Al_Hudayriyat,Bateen	2
Soleidae	Al_Hudayriyat,Seagrass	2
Engraulidae	Bateen,Mangrove	2
Monodactylidae	Bateen,Seagrass	2
Mugilidae	Bateen,Mangrove	2
Paralichthyidae	Bateen	1
Plotosidae	Bateen	1
Cyprinodontidae	Mangrove	1
**TAXA**	**Sites**	**N_sites**
Acanthopagrus sp._1	Al_Hudayriyat,Bateen,Mangrove,Seagrass	4
Acanthopagrus sp._2	Al_Hudayriyat,Bateen,Mangrove,Seagrass	4
Atherinomorus lacunosus	Al_Hudayriyat,Bateen,Mangrove,Seagrass	4
Carangidae	Al_Hudayriyat,Bateen,Mangrove,Seagrass	4
Clupeiformes_1	Al_Hudayriyat,Bateen,Mangrove,Seagrass	4
Epinephelus sp.	Al_Hudayriyat,Bateen,Mangrove,Seagrass	4
Favonigobius melanobranchus	Al_Hudayriyat,Bateen,Mangrove,Seagrass	4

Gerres sp._1	Al_Hudayriyat,Bateen,Mangrove,Seagrass	4
Gerres sp._3	Al_Hudayriyat,Bateen,Mangrove,Seagrass	4
Gnathanodon speciosus	Al_Hudayriyat,Bateen,Mangrove,Seagrass	4
Gobiidae_3	Al_Hudayriyat,Bateen,Mangrove,Seagrass	4
Hemiramphus sp._2	Al_Hudayriyat,Bateen,Mangrove,Seagrass	4
Lethrinus sp.	Al_Hudayriyat,Bateen,Mangrove,Seagrass	4
Lutjanus sp._2	Al_Hudayriyat,Bateen,Mangrove,Seagrass	4
Lutjanus sp._3	Al_Hudayriyat,Bateen,Mangrove,Seagrass	4
Monacanthidae	Al_Hudayriyat,Bateen,Mangrove,Seagrass	4
Nemipteridae_2	Al_Hudayriyat,Bateen,Mangrove,Seagrass	4
Pagellus sp.	Al_Hudayriyat,Bateen,Mangrove,Seagrass	4
Platycephalus indicus	Al_Hudayriyat,Bateen,Mangrove,Seagrass	4
Plectorhinchus sp._2	Al_Hudayriyat,Bateen,Mangrove,Seagrass	4
Pomacanthus maculosus	Al_Hudayriyat,Bateen,Mangrove,Seagrass	4
Rhabdosargus sp.	Al_Hudayriyat,Bateen,Mangrove,Seagrass	4
Scomberoides sp.	Al_Hudayriyat,Bateen,Mangrove,Seagrass	4
Scomberomorus commerson	Al_Hudayriyat,Bateen,Mangrove,Seagrass	4
Siganus sp.	Al_Hudayriyat,Bateen,Mangrove,Seagrass	4
Sphyraena sp._3	Al_Hudayriyat,Bateen,Mangrove,Seagrass	4
Terapon puta	Al_Hudayriyat,Bateen,Mangrove,Seagrass	4
Triacanthus biaculeatus	Al_Hudayriyat,Bateen,Mangrove,Seagrass	4
Tylosurus crocodilus	Al_Hudayriyat,Bateen,Mangrove,Seagrass	4
Upeneus sp._1	Al_Hudayriyat,Bateen,Mangrove,Seagrass	4
Valenciennea sp.	Al_Hudayriyat,Bateen,Mangrove,Seagrass	4
Apogonichthyoides sp.	Al_Hudayriyat,Mangrove,Seagrass	3
Blenniidae	Al_Hudayriyat,Bateen,Seagrass	3
Chanos chanos	Al_Hudayriyat,Mangrove,Seagrass	3
Cryptocentrus lutheri	Al_Hudayriyat,Mangrove,Seagrass	3
Gobiiformes_1	Al_Hudayriyat,Bateen,Seagrass	3
Leiognathidae	Al_Hudayriyat,Bateen,Seagrass	3
Nematalosa sp.	Al_Hudayriyat,Bateen,Seagrass	3
Nemipteridae_1	Al_Hudayriyat,Bateen,Seagrass	3
Platax sp.	Al_Hudayriyat,Bateen,Seagrass	3
Pseudochromis sp.	Al_Hudayriyat,Mangrove,Seagrass	3
Sillago sihama	Al_Hudayriyat,Bateen,Mangrove	3
Parupeneus sp.	Bateen,Mangrove,Seagrass	3
Clupeiformes_2	Al_Hudayriyat,Seagrass	2
Oreochromis sp._1	Al_Hudayriyat,Bateen	2
Pelates quadrilineatus	Al_Hudayriyat,Bateen	2
Pomadasys sp.	Al_Hudayriyat,Mangrove	2
Soleidae	Al_Hudayriyat,Seagrass	2
Sphyraena sp._1	Al_Hudayriyat,Mangrove	2
Sphyraena sp._2	Al_Hudayriyat,Mangrove	2
Upeneus sp._2	Al_Hudayriyat,Seagrass	2
Encrasicholina heteroloba	Bateen,Mangrove	2
Gerres sp._2	Bateen,Seagrass	2
Monodactylus argenteus	Bateen,Seagrass	2
Alepes sp._1	Al_Hudayriyat	1

Alepes sp._2	Al_Hudayriyat	1
Apogonidae	Al_Hudayriyat	1
Diplodus kotschyi	Al_Hudayriyat	1
Oreochromis sp._2	Al_Hudayriyat	1
Plectorhinchus sp._1	Al_Hudayriyat	1
Rastrelliger kanagurta	Al_Hudayriyat	1
Mugilidae	Bateen	1
Paralichthyidae	Bateen	1
Plotosus lineatus	Bateen	1
Aphaniops stoliczkanus	Mangrove	1
Gobiidae_2	Mangrove	1
Hemiramphus sp._1	Mangrove	1
Lutjanus sp._1	Mangrove	1
Moolgarda seheli	Mangrove	1
Perciformes	Mangrove	1
Sardinella sp._1	Mangrove	1
Sardinella sp._2	Mangrove	1
Gobiidae_1	Seagrass	1
Gobiidae_4	Seagrass	1
Gobiiformes_2	Seagrass	1
