# Supplementary material for: First eDNA-based monitoring of marine vertebrates in the urban marine ecosystem of Abu Dhabi: Mangrove and seagrass beds as biodiversity reservoirs?
Source: PLoS One. 2026 Aug 4;21(8):e0352249. doi: 10.1371/journal.pone.0352249 (PMC13436779; doi:10.1371/journal.pone.0352249)

Appendix 2: Matrix showing turnover (above the diagonal) and nestedness (below the diagonal) for taxonomic and phylogenetic beta-diversity

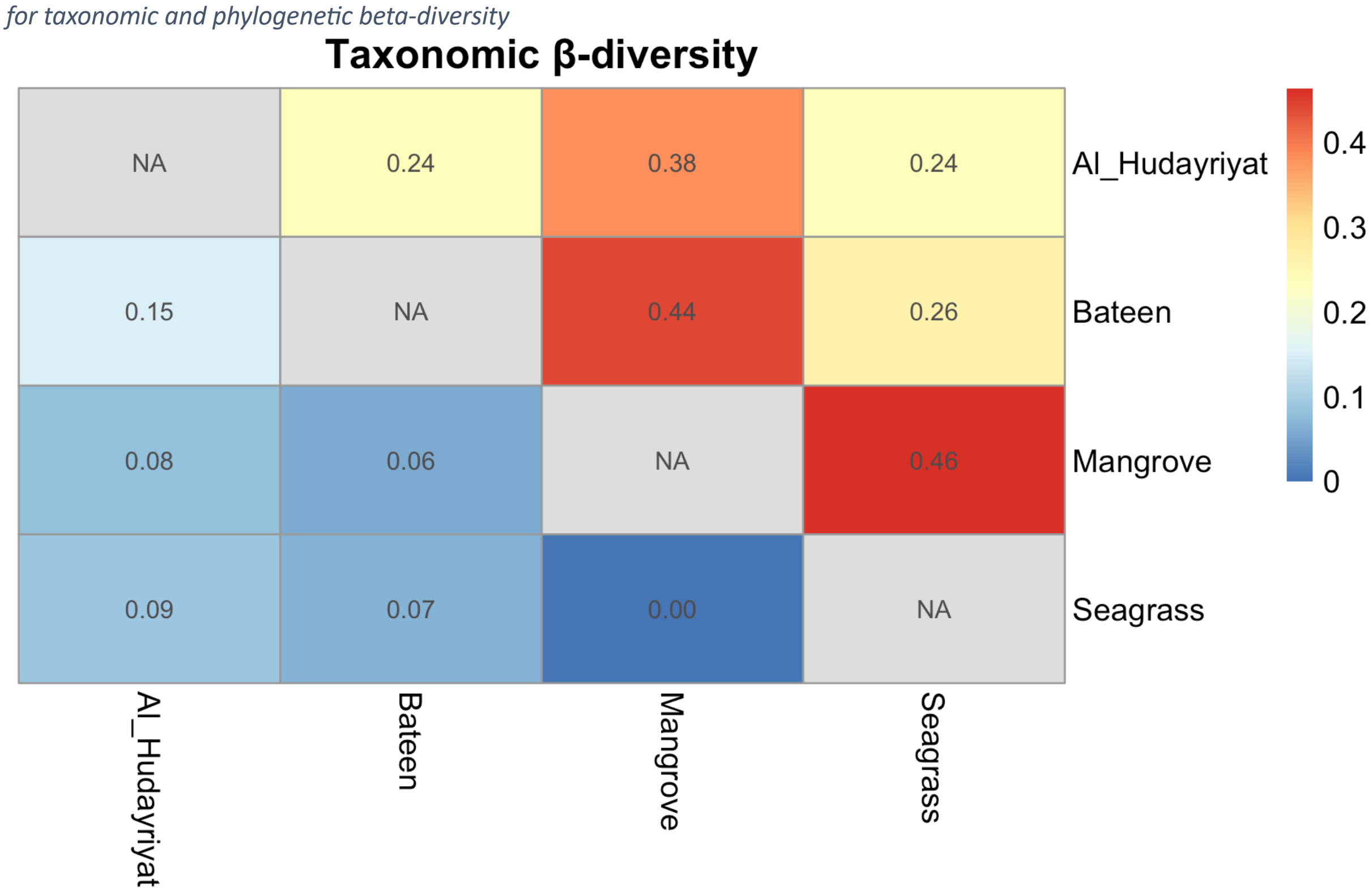

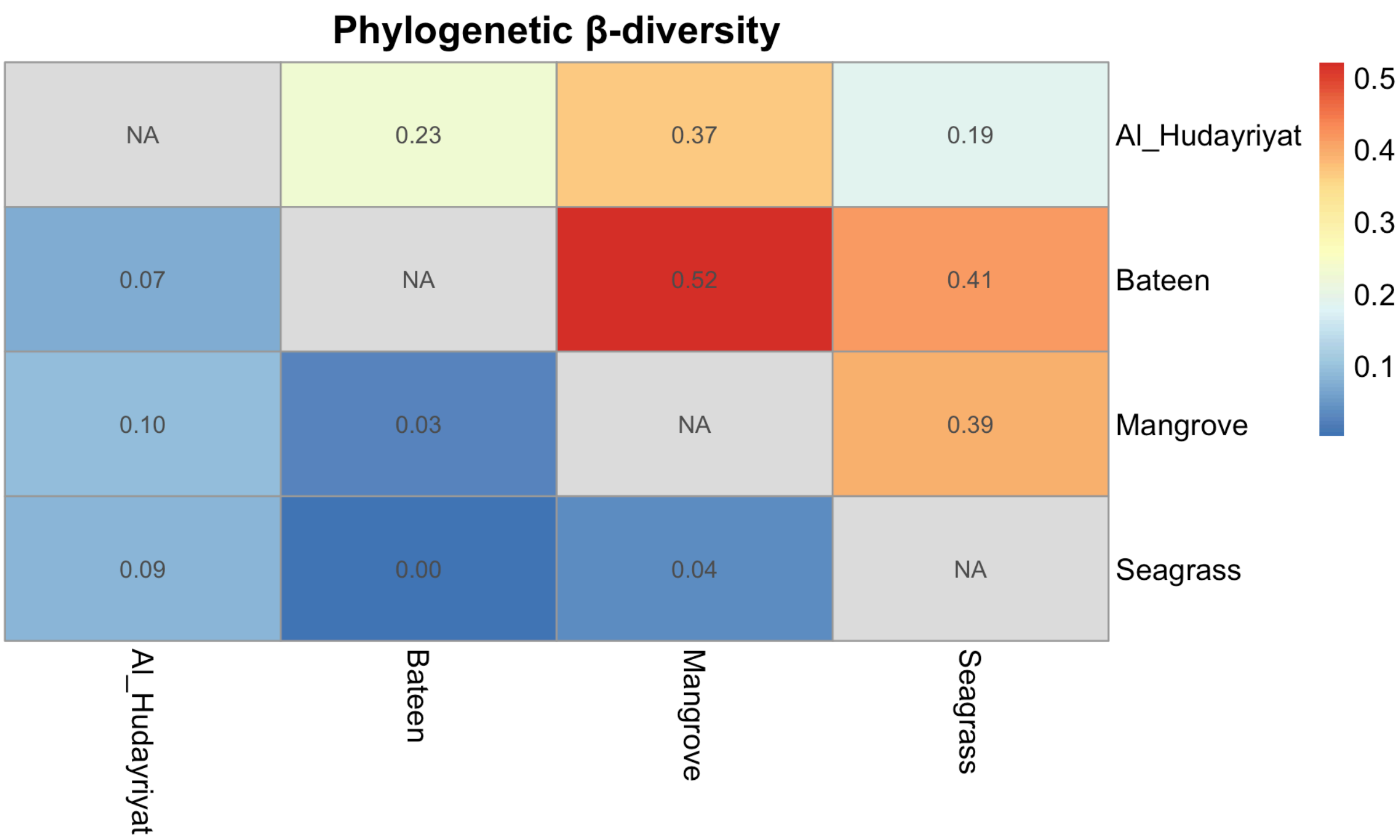

Supplement: Supplementary file 2 [file pone.0352249.s002.pdf]
