# Supplementary material for: First eDNA-based monitoring of marine vertebrates in the urban marine ecosystem of Abu Dhabi: Mangrove and seagrass beds as biodiversity reservoirs?
Source: PLoS One. 2026 Aug 4;21(8):e0352249. doi: 10.1371/journal.pone.0352249 (PMC13436779; doi:10.1371/journal.pone.0352249)
Supplement: Supplementary file 5 [file pone.0352249.s003.docx]

Overall, this paper presents a tidy eDNA metabarcoding exploration of four sites off the coast of Abu Dhabi. The methods need to be clarified in a few places, but the paper is an interesting inventory of species that can be used as a baseline for future research.

In addition to specific methodological details which must be included before publication (ie, identity threshold in OBItools and reference database curation methods), I recommend the authors cut down/streamline a little bit of the introduction of the region as a whole and instead focus on the actual sampling sites to introduce readers to your hypotheses. IE, how are these sites different from each other? What role does each ecosystem play in harboring diversity in UAE waters, and how does that affect your expectations of what you’re going to find? What patterns have previous, non-eDNA surveys found?

Basically, explain the biological reasoning behind why these four sites were picked, and what you expect to find. I say this because I think you have a really cool setup here and it can answer interesting questions, and I think it should be highlighted. I also think it will both contextualize and help with the flow of the discussion by giving you something to call back to.

Below are my line-specific edits.

Line specific review:

67 – Low species diversity compared to the same habitats in other, less extreme waters, right? The introduction of mangroves and coral reefs, famous for their high diversity, makes this sentence a bit hard to follow.

74 – climate projects for what region specifically? The whole paragraph varies between “UAE waters” and what I think is referring to the whole Arabian gulf. Probably an easy change to clarify.

164 – MOTUs were checked “meticulously by hand”. Checked against what? A taxonomic database (ie, BLAST, a local database, BOLD, etc.)? Or checked for a certain % identity? This is a key step for your analysis, so please be as accurate as possible. I understand you used OBITools – please be more descriptive. I assume you used ecotag? What did you set your % identity cutoff as (and why?) What did you use as your reference sequences? How did you develop that reference database? Did you use ecoPCR? If it was EMBL, which version, accessed on what date? If it was hand-curated, did you include common contaminants (ie, human, domestic animals)? Are you confident in how robust your database is?

166 – So is a “taxonomic redundancy” a case where two MOTUs were above your % identity similarity threshold, yet still assigned to the same species? We need far more detail here about how you assigned MOTUs to species.

187 – Missing word – “Unifrac also allowed [us?] to decompose…”

207-219 – This is a pretty long description of taxa that are available in a table. I think given the length of the paper, this paragraph can be removed *unless* any of these were new records. I do appreciate noting that one of your taxa was confirmed with visual observation, though.

238 – You vary between italics and regular face for family names. Just a formatting note.

307 – Typo: “Trough” for “through”

311- It isn’t a great message to just blame your insufficient reference database on the “current limitations of reference databases.” Doing it yourself is always an option – a curated reference database is always best, and producing the sequences for projects like these is a primary way the sequencing databases grow. Producing location-relevant sequences is something every metabarcoding project should consider, especially one developing a forward-looking protocol for monitoring a specific region. I appreciate that this project probably was not of large enough scope to produce any sequencing, but with the cheapness and availability of Sanger sequencing, a sentence about why the project did not/could not produce relevant sequences for known teleosts might be appropriate.

312 – I also want to ask – Is the failure to resolve at the species level for so many teleosts exclusively because they aren’t in the database, or is there evidence that some of them are not variable/distinct enough at these primers to be resolved? If so, please mention it, because it represents a different kind of limitation for this study into the future.

320 – Another formatting note, you vary whether you spell out or use numbers for numbers <10, in the results you used numerals, but here you spell out. The PLoS editors will have guidance for this, no biggie.

377 – Typo: “Areas” for “Area”.
