# Supplementary material for: First eDNA-based monitoring of marine vertebrates in the urban marine ecosystem of Abu Dhabi: Mangrove and seagrass beds as biodiversity reservoirs?
Source: PLoS One. 2026 Aug 4;21(8):e0352249. doi: 10.1371/journal.pone.0352249 (PMC13436779; doi:10.1371/journal.pone.0352249)
Supplement: Peer review history

# Author comment: Author response to Decision Letter 0

**DOI:** 10.1371/journal.pone.0352249.r001

**Transfer Alert**

This paper was transferred from another journal. As a result, its full editorial history (including decision letters, peer reviews and author responses) may not be present.

18 Feb 2026

# Peer review: Decision Letter 0

**DOI:** 10.1371/journal.pone.0352249.r002

**Authors:** Petr Heneberg

30 Apr 2026

PONE-D-26-08592

First eDNA-Based Monitoring of Marine Vertebrates in the Urban Marine Ecosystem of Abu Dhabi: Mangrove and Seagrass beds as biodiversity reservoirs?

PLOS One

Dear Dr. Jung,

Thank you for submitting your manuscript to PLOS ONE. After careful consideration, we feel that it has merit but does not fully meet PLOS ONE’s publication criteria as it currently stands. Therefore, we invite you to submit a revised version of the manuscript that addresses the points raised during the review process.

Please submit your revised manuscript by Jun 14 2026 11:59PM. If you will need more time than this to complete your revisions, please reply to this message or contact the journal office at plosone@plos.org. When you're ready to submit your revision, log on to https://www.editorialmanager.com/pone/ and select the 'Submissions Needing Revision' folder to locate your manuscript file.

Please include the following items when submitting your revised manuscript:

- A letter that responds to each point raised by the academic editor and reviewer(s). You should upload this letter as a separate file labeled 'Response to Reviewers'.
- A marked-up copy of your manuscript that highlights changes made to the original version. You should upload this as a separate file labeled 'Revised Manuscript with Track Changes'.
- An unmarked version of your revised paper without tracked changes. You should upload this as a separate file labeled 'Manuscript'.

If you would like to make changes to your financial disclosure, please include your updated statement in your cover letter. Guidelines for resubmitting your figure files are available below the reviewer comments at the end of this letter.

If applicable, we recommend that you deposit your laboratory protocols in protocols.io to enhance the reproducibility of your results. Protocols.io assigns your protocol its own identifier (DOI) so that it can be cited independently in the future. For instructions see: https://journals.plos.org/plosone/s/submission-guidelines#loc-laboratory-protocols. Additionally, PLOS ONE offers an option for publishing peer-reviewed Lab Protocol articles, which describe protocols hosted on protocols.io. Read more information on sharing protocols at https://plos.org/protocols?utm_medium=editorial-email&utm_source=authorletters&utm_campaign=protocols.

As the corresponding author, your ORCID iD is verified in the submission system and will appear in the published article. PLOS supports the use of ORCID, and we encourage all coauthors to register for an ORCID iD and use it as well. Please encourage your coauthors to verify their ORCID iD within the submission system before final acceptance, as unverified ORCID iDs will not appear in the published article. *Only*  the individual author can complete the verification step; PLOS staff *cannot*  verify ORCID iDs on behalf of authors.

We look forward to receiving your revised manuscript.

Kind regards,

Petr Heneberg

Academic Editor

PLOS One

Journal requirements:

When submitting your revision, we need you to address these additional requirements.

1. Please ensure that your manuscript meets PLOS ONE's style requirements, including those for file naming. The PLOS ONE style templates can be found at

https://journals.plos.org/plosone/s/file?id=wjVg/PLOSOne_formatting_sample_main_body.pdf and

https://journals.plos.org/plosone/s/file?id=ba62/PLOSOne_formatting_sample_title_authors_affiliations.pdf

2. In your Methods section, please provide additional information regarding the permits you obtained for the work. Please ensure you have included the full name of the authority that approved the field site access and, if no permits were required, a brief statement explaining why.

3. Thank you for stating the following financial disclosure:

“This study was found by Sorbonne University Abu Dhabi. The vessels and crews were provided by the Abu Dhabi Environment Agency.”

Please state what role the funders took in the study.  If the funders had no role, please state: "The funders had no role in study design, data collection and analysis, decision to publish, or preparation of the manuscript."

If this statement is not correct you must amend it as needed.

Please include this amended Role of Funder statement in your cover letter; we will change the online submission form on your behalf.

4. Please amend either the abstract on the online submission form (via Edit Submission) or the abstract in the manuscript so that they are identical.

5. We note that Figure 1 in your submission contain [map/satellite] images which may be copyrighted. All PLOS content is published under the Creative Commons Attribution License (CC BY 4.0), which means that the manuscript, images, and Supporting Information files will be freely available online, and any third party is permitted to access, download, copy, distribute, and use these materials in any way, even commercially, with proper attribution. For these reasons, we cannot publish previously copyrighted maps or satellite images created using proprietary data, such as Google software (Google Maps, Street View, and Earth). For more information, see our copyright guidelines: http://journals.plos.org/plosone/s/licenses-and-copyright.

We require you to either (1) present written permission from the copyright holder to publish these figures specifically under the CC BY 4.0 license, or (2) remove the figures from your submission:

1. You may seek permission from the original copyright holder of Figure 1 to publish the content specifically under the CC BY 4.0 license.

We recommend that you contact the original copyright holder with the Content Permission Form (http://journals.plos.org/plosone/s/file?id=7c09/content-permission-form.pdf) and the following text:

“I request permission for the open-access journal PLOS ONE to publish XXX under the Creative Commons Attribution License (CCAL) CC BY 4.0 (http://creativecommons.org/licenses/by/4.0/). Please be aware that this license allows unrestricted use and distribution, even commercially, by third parties. Please reply and provide explicit written permission to publish XXX under a CC BY license and complete the attached form.”

Please upload the completed Content Permission Form or other proof of granted permissions as an "Other" file with your submission.

In the figure caption of the copyrighted figure, please include the following text: “Reprinted from [ref] under a CC BY license, with permission from [name of publisher], original copyright [original copyright year].”

2. If you are unable to obtain permission from the original copyright holder to publish these figures under the CC BY 4.0 license or if the copyright holder’s requirements are incompatible with the CC BY 4.0 license, please either i) remove the figure or ii) supply a replacement figure that complies with the CC BY 4.0 license. Please check copyright information on all replacement figures and update the figure caption with source information. If applicable, please specify in the figure caption text when a figure is similar but not identical to the original image and is therefore for illustrative purposes only.

The following resources for replacing copyrighted map figures may be helpful:

USGS National Map Viewer (public domain): http://viewer.nationalmap.gov/viewer/

The Gateway to Astronaut Photography of Earth (public domain): http://eol.jsc.nasa.gov/sseop/clickmap/

Maps at the CIA (public domain): https://www.cia.gov/library/publications/the-world-factbook/index.html and https://www.cia.gov/library/publications/cia-maps-publications/index.html

NASA Earth Observatory (public domain): http://earthobservatory.nasa.gov/

Landsat: http://landsat.visibleearth.nasa.gov/

USGS EROS (Earth Resources Observatory and Science (EROS) Center) (public domain): http://eros.usgs.gov/#

Natural Earth (public domain): http://www.naturalearthdata.com/

6. Please include captions for your Supporting Information files at the end of your manuscript, and update any in-text citations to match accordingly. Please see our Supporting Information guidelines for more information: http://journals.plos.org/plosone/s/supporting-information.

If the reviewer comments include a recommendation to cite specific previously published works, please review and evaluate these publications to determine whether they are relevant and should be cited. There is no requirement to cite these works unless the editor has indicated otherwise.

Additional Editor Comments:

Please note that failing to reflect the raised comments of the reviewers on the methodological approaches used (missing controls) would result in manuscript rejection. If you need more time for the revision (to perform additional experiments), please do not hesitate to apply for the extension of the revision deadline.

[Note: HTML markup is below. Please do not edit.]

Reviewers' comments:

Reviewer's Responses to Questions

**Comments to the Author**

1. Is the manuscript technically sound, and do the data support the conclusions?

The manuscript must describe a technically sound piece of scientific research with data that supports the conclusions. Experiments must have been conducted rigorously, with appropriate controls, replication, and sample sizes. The conclusions must be drawn appropriately based on the data presented.

Reviewer #1: No

Reviewer #2: Yes

**********

2. Has the statistical analysis been performed appropriately and rigorously?

Reviewer #1: No

Reviewer #2: Yes

**********

3. Have the authors made all data underlying the findings in their manuscript fully available?

The PLOS Data policy requires authors to make all data underlying the findings described in their manuscript fully available without restriction, with rare exception (please refer to the Data Availability Statement in the manuscript PDF file). The data should be provided as part of the manuscript or its supporting information, or deposited to a public repository. For example, in addition to summary statistics, the data points behind means, medians and variance measures should be available. If there are restrictions on publicly sharing data—e.g. participant privacy or use of data from a third party—those must be specified.

Reviewer #1: Yes

Reviewer #2: Yes

**********

4. Is the manuscript presented in an intelligible fashion and written in standard English?

PLOS ONE does not copyedit accepted manuscripts, so the language in submitted articles must be clear, correct, and unambiguous. Any typographical or grammatical errors should be corrected at revision, so please note any specific errors here.

Reviewer #1: Yes

Reviewer #2: Yes

**********

5. Review Comments to the Author

Please use the space provided to explain your answers to the questions above. You may also include additional comments for the author, including concerns about dual publication, research ethics, or publication ethics. (Please upload your review as an attachment if it exceeds 20,000 characters)

Reviewer #1: This manuscript presents an environmental DNA (eDNA) metabarcoding survey of marine vertebrate biodiversity across four urban coastal habitats in Abu Dhabi. While the study has the potential to generate interesting insights, several methodological limitations, some of which (in my opinion) may not be addressable at this stage, render it unsuitable for publication in PLOS ONE. First, it seems like field blanks were not included in the surveys, there is no mention of DNA extraction controls, or PCR negative controls. This raises concerns about how potential contamination during both field sampling and laboratory processing was assessed and mitigated. This represents a major shortcoming of the study. Second, the sampling design is insufficient to support robust ecological inference. The study is based on only four sites sampled over two days, with duplicate filtrations. The lack of temporal replication, limited spatial replication within habitat types, and the absence of a clear distinction between technical and biological replicates weaken the strength of the conclusions. Third, only approximately 25% of taxa were resolved to the species level. Although this limitation is acknowledged, it is not adequately integrated into the interpretation of the results. Fourth, statistical analyses comparing richness/diversity indices across sites should account for spatial autocorrelation, otherwise the authors need to prove with strong arguments that there is no spatial autocorrelation.

Minor comments:

Lines 37-40: Please rephrase. The words “worrying” and “with regard to” are not appropriate in scientific writing.

Lines 41-42: The word “inventory” assumes that the authors have done a very comprehensive eDNA survey. Also, doing a one-off survey does not result into a marine vertebrate inventory. I am not sure if four sites count as comprehensive. I recommend toning down this claim. Also, please include the genes targeted in this eDNA survey, as this info can provide an idea of how comprehensive were these surveys.

Lines 51-55: this paragraph should come after the introductory sentences. Also, please rephrase.

Lines 81-82: This seems repetitive, I recommend deleting.

Lines 115-116: Please tone down this claim.

Line 116: What does “Using a standardized workflow of water sampling” mean, please give a bit more detail about who standardized this method.

Lines 127-131: I suggest removing this, as it has already been explained in the Introduction.

Figure 1 can be improved. Please add a reference map and a better delineation of the sampling sites. By looking at this map, I don’t think these scattered four sites would give an “inventory” of the marine vertebrates of Abu Dhabi.

Lines 146-150: I understand that at each site, samples were collected along two transects? If this is the case, please say so and provide the GPS points and length of the transects. Also, please describe briefly the protocol cited (ref #40).

Lines 151-152: what about field blanks? This is such an important aspect in eDNA fieldwork. I am afraid that there is no way of proving that the results of this study are not result of sample contamination if there were no field blanks. Also, there is no mention of DNA extractions and extraction controls, or of PCR negative controls.

Lines 156-158: I think the right approach to detect “all” marine vertebrates would have been to sequence more than one gene. There are fish, elasmobranchs, mammals and birds’ specific universal assays available. The choice of only one gene needs to be justified.

Lines 159-161: The bioinformatics need much more detail than three lines. Include filtering parameters, and software/version used.

Lines 104-248: It is unclear how the authors can demonstrate that the detected species originate solely from the sampled environment rather than being influenced by potential contamination during field collection or laboratory processing.

Line 250: I am not sure if this is the right statistical analysis. I assume there is a strong spatial autocorrelation between sites, and this needs to be taken into account for statistical analyses. Without proving that data is not spatially autocorrelated, statements such as “Taxonomic dissimilarity was generally moderate to low across site” could be arising from incorrect analysis.

Reviewer #2: Overall, this paper presents a tidy eDNA metabarcoding exploration of four sites off the coast of Abu Dhabi. The methods need to be clarified in a few places, but the paper is an interesting inventory of species that can be used as a baseline for future research.

In addition to specific methodological details which must be included before publication (ie, identity threshold in OBItools and reference database curation methods), I recommend the authors cut down/streamline a little bit of the introduction of the region as a whole and instead focus on the actual sampling sites to introduce readers to your hypotheses. IE, how are these sites different from each other? What role does each ecosystem play in harboring diversity in UAE waters, and how does that affect your expectations of what you’re going to find? What patterns have previous, non-eDNA surveys found?

Basically, explain the biological reasoning behind why these four sites were picked, and what you expect to find. I say this because I think you have a really cool setup here and it can answer interesting questions, and I think it should be highlighted. I also think it will both contextualize and help with the flow of the discussion by giving you something to call back to.

Below are my line-specific edits.

Line specific review:

67 – Low species diversity compared to the same habitats in other, less extreme waters, right? The introduction of mangroves and coral reefs, famous for their high diversity, makes this sentence a bit hard to follow.

74 – climate projects for what region specifically? The whole paragraph varies between “UAE waters” and what I think is referring to the whole Arabian gulf. Probably an easy change to clarify.

164 – MOTUs were checked “meticulously by hand”. Checked against what? A taxonomic database (ie, BLAST, a local database, BOLD, etc.)? Or checked for a certain % identity? This is a key step for your analysis, so please be as accurate as possible. I understand you used OBITools – please be more descriptive. I assume you used ecotag? What did you set your % identity cutoff as (and why?) What did you use as your reference sequences? How did you develop that reference database? Did you use ecoPCR? If it was EMBL, which version, accessed on what date? If it was hand-curated, did you include common contaminants (ie, human, domestic animals)? Are you confident in how robust your database is?

166 – So is a “taxonomic redundancy” a case where two MOTUs were above your % identity similarity threshold, yet still assigned to the same species? We need far more detail here about how you assigned MOTUs to species.

187 – Missing word – “Unifrac also allowed [us?] to decompose…”

207-219 – This is a pretty long description of taxa that are available in a table. I think given the length of the paper, this paragraph can be removed unless any of these were new records. I do appreciate noting that one of your taxa was confirmed with visual observation, though.

238 – You vary between italics and regular face for family names. Just a formatting note.

307 – Typo: “Trough” for “through”

311- It isn’t a great message to just blame your insufficient reference database on the “current limitations of reference databases.” Doing it yourself is always an option – a curated reference database is always best, and producing the sequences for projects like these is a primary way the sequencing databases grow. Producing location-relevant sequences is something every metabarcoding project should consider, especially one developing a forward-looking protocol for monitoring a specific region. I appreciate that this project probably was not of large enough scope to produce any sequencing, but with the cheapness and availability of Sanger sequencing, a sentence about why the project did not/could not produce relevant sequences for known teleosts might be appropriate.

312 – I also want to ask – Is the failure to resolve at the species level for so many teleosts exclusively because they aren’t in the database, or is there evidence that some of them are not variable/distinct enough at these primers to be resolved? If so, please mention it, because it represents a different kind of limitation for this study into the future.

320 – Another formatting note, you vary whether you spell out or use numbers for numbers <10, in the results you used numerals, but here you spell out. The PLoS editors will have guidance for this, no biggie.

377 – Typo: “Areas” for “Area”.

**********

6. PLOS authors have the option to publish the peer review history of their article (what does this mean?). If published, this will include your full peer review and any attached files.

If you choose “no”, your identity will remain anonymous but your review may still be made public.

**Do you want your identity to be public for this peer review?**  For information about this choice, including consent withdrawal, please see our Privacy Policy.

Reviewer #1: **Yes:**  Cecilia Villacorta-Rath

Reviewer #2: No

**********

[NOTE: If reviewer comments were submitted as an attachment file, they will be attached to this email and accessible via the submission site. Please log into your account, locate the manuscript record, and check for the action link "View Attachments". If this link does not appear, there are no attachment files.]

To ensure your figures meet our technical requirements, please review our figure guidelines: https://journals.plos.org/plosone/s/figures

You may also use PLOS’s free figure tool, NAAS, to help you prepare publication quality figures: https://journals.plos.org/plosone/s/figures#loc-tools-for-figure-preparation.

NAAS will assess whether your figures meet our technical requirements by comparing each figure against our figure specifications.

Attachment Submitted filename: jung_et_al_review.docx

# Author comment: Author response to Decision Letter 1

**DOI:** 10.1371/journal.pone.0352249.r003

4 Jun 2026

Jean-Luc Jung

Corresponding Author

Professor, ISYEB, Muséum national d’Histoire naturelle, France

SAFIR- Sorbonne Université Abu Dhabi

jean-luc.jung@mnhn.fr

Dinard, the 3 June 2026

Dr Petr Heneberg

Academic Editor

PLOS One

Dear Editor,

We would like to thank you and the reviewers for your work in evaluating our publication. We have carefully read and considered the evaluations provided. We have thoroughly addressed each point raised by the reviewers and we have revised the manuscript accordingly. We upload today a “Revised Manuscript with Track Changes” file, and a clean file with all modification accepted named “Manuscript”.

Our detailed responses to all comments are provided in the following pages; all our responses are highlighted in blue to facilitate the reading.

Yours sincerely,

Jean-Luc Jung, on the behalf of all Authors

PONE-D-26-08592

First eDNA-Based Monitoring of Marine Vertebrates in the Urban Marine Ecosystem of Abu Dhabi: Mangrove and Seagrass beds as biodiversity reservoirs?

PLOS One

Dear Dr. Jung,

Thank you for submitting your manuscript to PLOS ONE. After careful consideration, we feel that it has merit but does not fully meet PLOS ONE’s publication criteria as it currently stands. Therefore, we invite you to submit a revised version of the manuscript that addresses the points raised during the review process.

Please submit your revised manuscript by Jun 14 2026 11:59PM. If you will need more time than this to complete your revisions, please reply to this message or contact the journal office at plosone@plos.org. When you're ready to submit your revision, log on to https://www.editorialmanager.com/pone/and select the 'Submissions Needing Revision' folder to locate your manuscript file.

Please include the following items when submitting your revised manuscript:

• A letter that responds to each point raised by the academic editor and reviewer(s). You should upload this letter as a separate file labeled 'Response to Reviewers'.

• A marked-up copy of your manuscript that highlights changes made to the original version. You should upload this as a separate file labeled 'Revised Manuscript with Track Changes'.

• An unmarked version of your revised paper without tracked changes. You should upload this as a separate file labeled 'Manuscript'.

If you would like to make changes to your financial disclosure, please include your updated statement in your cover letter. Guidelines for resubmitting your figure files are available below the reviewer comments at the end of this letter.

If applicable, we recommend that you deposit your laboratory protocols in protocols.io to enhance the reproducibility of your results. Protocols.io assigns your protocol its own identifier (DOI) so that it can be cited independently in the future. For instructions see: https://journals.plos.org/plosone/s/submission-guidelines#loc-laboratory-protocols. Additionally, PLOS ONE offers an option for publishing peer-reviewed Lab Protocol articles, which describe protocols hosted on protocols.io. Read more information on sharing protocols at https://plos.org/protocols?utm_medium=editorial-email&utm_source=authorletters&utm_campaign=protocols.

As the corresponding author, your ORCID iD is verified in the submission system and will appear in the published article. PLOS supports the use of ORCID, and we encourage all coauthors to register for an ORCID iD and use it as well. Please encourage your coauthors to verify their ORCID iD within the submission system before final acceptance, as unverified ORCID iDs will not appear in the published article. Only the individual author can complete the verification step; PLOS staff cannot verify ORCID iDs on behalf of authors.

We look forward to receiving your revised manuscript.

Kind regards,

Petr Heneberg

Academic Editor

PLOS One

Journal requirements:

When submitting your revision, we need you to address these additional requirements.

1. Please ensure that your manuscript meets PLOS ONE's style requirements, including those for file naming. The PLOS ONE style templates can be found at

https://journals.plos.org/plosone/s/file?id=wjVg/PLOSOne_formatting_sample_main_body.pdf and

https://journals.plos.org/plosone/s/file?id=ba62/PLOSOne_formatting_sample_title_authors_affiliations.pdf

Done. The manuscript is now following PLOS ONE’s style requirements

2. In your Methods section, please provide additional information regarding the permits you obtained for the work. Please ensure you have included the full name of the authority that approved the field site access and, if no permits were required, a brief statement explaining why.

Done. The permit details are listed in the manuscript

3. Thank you for stating the following financial disclosure:

“This study was found by Sorbonne University Abu Dhabi. The vessels and crews were provided by the Abu Dhabi Environment Agency.”

Please state what role the funders took in the study. If the funders had no role, please state: "The funders had no role in study design, data collection and analysis, decision to publish, or preparation of the manuscript."

If this statement is not correct you must amend it as needed.

Please include this amended Role of Funder statement in your cover letter; we will change the online submission form on your behalf.

We include this statement in the cover letter

The Environment Agency provided advice on the selection of sites and supplied the vessel used for sampling, as well as the crew. Apart from that, the funders had no role in study design, data collection and analysis, decision to publish, or preparation of the manuscript.

4. Please amend either the abstract on the online submission form (via Edit Submission) or the abstract in the manuscript so that they are identical.

Done

5. We note that Figure 1 in your submission contain [map/satellite] images which may be copyrighted. All PLOS content is published under the Creative Commons Attribution License (CC BY 4.0), which means that the manuscript, images, and Supporting Information files will be freely available online, and any third party is permitted to access, download, copy, distribute, and use these materials in any way, even commercially, with proper attribution. For these reasons, we cannot publish previously copyrighted maps or satellite images created using proprietary data, such as Google software (Google Maps, Street View, and Earth). For more information, see our copyright guidelines: http://journals.plos.org/plosone/s/licenses-and-copyright.

We require you to either (1) present written permission from the copyright holder to publish these figures specifically under the CC BY 4.0 license, or (2) remove the figures from your submission:

Figure 1 has been amended and now complies with the required licensing rules

6. Please include captions for your Supporting Information files at the end of your manuscript, and update any in-text citations to match accordingly. Please see our Supporting Information guidelines for more information: http://journals.plos.org/plosone/s/supporting-information.

If the reviewer comments include a recommendation to cite specific previously published works, please review and evaluate these publications to determine whether they are relevant and should be cited. There is no requirement to cite these works unless the editor has indicated otherwise.

Done

Additional Editor Comments:

Please note that failing to reflect the raised comments of the reviewers on the methodological approaches used (missing controls) would result in manuscript rejection. If you need more time for the revision (to perform additional experiments), please do not hesitate to apply for the extension of the revision deadline.

[Note: HTML markup is below. Please do not edit.]

We hope that, in this new version, we have provided convincing answers to all the reviewers’ questions and included all the additional information and clarifications requested.

Reviewers' comments:

Reviewer's Responses to Questions

Comments to the Author

1. Is the manuscript technically sound, and do the data support the conclusions?

The manuscript must describe a technically sound piece of scientific research with data that supports the conclusions. Experiments must have been conducted rigorously, with appropriate controls, replication, and sample sizes. The conclusions must be drawn appropriately based on the data presented.

Reviewer #1: No

Reviewer #2: Yes

2. Has the statistical analysis been performed appropriately and rigorously?

Reviewer #1: No

Reviewer #2: Yes

3. Have the authors made all data underlying the findings in their manuscript fully available?

The PLOS Data policy requires authors to make all data underlying the findings described in their manuscript fully available without restriction, with rare exception (please refer to the Data Availability Statement in the manuscript PDF file). The data should be provided as part of the manuscript or its supporting information, or deposited to a public repository. For example, in addition to summary statistics, the data points behind means, medians and variance measures should be available. If there are restrictions on publicly sharing data—e.g. participant privacy or use of data from a third party—those must be specified.

Reviewer #1: Yes

Reviewer #2: Yes

4. Is the manuscript presented in an intelligible fashion and written in standard English?

PLOS ONE does not copyedit accepted manuscripts, so the language in submitted articles must be clear, correct, and unambiguous. Any typographical or grammatical errors should be corrected at revision, so please note any specific errors here.

Reviewer #1: Yes

Reviewer #2: Yes

5. Review Comments to the Author

Please use the space provided to explain your answers to the questions above. You may also include additional comments for the author, including concerns about dual publication, research ethics, or publication ethics. (Please upload your review as an attachment if it exceeds 20,000 characters)

Reviewer #1: This manuscript presents an environmental DNA (eDNA) metabarcoding survey of marine vertebrate biodiversity across four urban coastal habitats in Abu Dhabi. While the study has the potential to generate interesting insights, several methodological limitations, some of which (in my opinion) may not be addressable at this stage, render it unsuitable for publication in PLOS ONE. First, it seems like field blanks were not included in the surveys, there is no mention of DNA extraction controls, or PCR negative controls. This raises concerns about how potential contamination during both field sampling and laboratory processing was assessed and mitigated. This represents a major shortcoming of the study.

We thank the reviewer for raising this important point regarding contamination controls.

To monitor any potential laboratory contamination, negative controls were included during the DNA extraction and the PCR amplification and were sequenced in parallel with the samples. These controls were systematically inspected during the bioinformatic analysis, and only human and domestic animals DNA was detected in the controls. This has been added in the new version of the manuscript.

Regarding field negative controls, we agree with the reviewer that they are highly recommended, and indeed mandatory when sampling involves open filtration systems or reusable equipment that is decontaminated between samples (e.g., Bruce et al., 2021, https://doi.org/10.3897/ab.e68634).

However, these conditions do not apply to our sampling protocol. All materials in contact with water were single-use, and filtration was carried out using enclosed, gamma-sterilized capsule filters that were opened only in the field, at the moment of filtration. The openings have the same diameter as the pipe, approximately 0.5 cm in diameter.

The only DNA that could enter the filter is therefore the eDNA present at the sampling site itself. Immediately after filtration, each filter was sealed and stored individually before being transported to the laboratory for DNA extraction. A negative control would in addition have required transporting 30 liters of sterile water onto the ship, resulting in significant logistical challenges.

Under these conditions, and considering the risk of field contamination to be extremely low because (i) of the characteristics of our filter and (ii) the experimental precautions we take, we believe that a field negative control would not have provided additional information relevant to the interpretation of our results.

Nevertheless, we totally agree with the reviewer that negative controls are of major importance in eDNA analysis, even though, under our experimental conditions, field-contamination is highly unlikely (all the required Laboratory controls, - DNA extraction controls and PCR negative controls - were performed.

Nevertheless, before conducting our next experiments, we will consider the best way to implement these controls.

Second, the sampling design is insufficient to support robust ecological inference. The study is based on only four sites sampled over two days, with duplicate filtrations. The lack of temporal replication, limited spatial replication within habitat types, and the absence of a clear distinction between technical and biological replicates weaken the strength of the conclusions.

We acknowledge that additional spatial and temporal replication would have strengthened the study, however, we would like to clarify our sampling strategy, which was deliberately designed to maximize species detectability rather than to maximize the number of discrete sampling units.

Increasing sample volume is recognized to enhance the sensitivity of eDNA survey (eg Takahashi et al, 2023, http://dx.doi.org/10.1016/j.scitotenv.2023.162322).

Unlike conventional point sampling, our protocol relied on transect-based filtration, during which the pump was moved along the transect to cover a large surface area. Each transect therefore integrates eDNA signal over a much larger spatial footprint than a single point sample, which compensates for the limited number of discrete sampling stations.

In addition, each site was sampled with two field replicates of 30 L each (60 L per site in total), which is substantially higher than the volumes typically reported in marine eDNA studies. Kawakami et al., (2023, https://doi.org/10.1002/ece3.9921) showed that, in open-ocean conditions, species accumulation curves rarely saturate and that detection of the full fish community requires either tens of replicates of 1L or a substantial filtration volume. Our design explicitly favored the second strategy: increasing the filtered volume per site to enhance detection probability, given the known stochasticity of eDNA capture in marine environments.

We fully agree that a design with more sites and more temporal replicates would have been ideal. However, we chose to prioritize a sampling strategy that maximizes the detectability of species at each site (transect filtration and large filtered volumes) rather than increasing the number of replicates at the cost of lower detection probability per unit. We believe this trade-off is justified for the ecological questions addressed here.

Our manuscript describes an initial experimental campaign, the results of which we believe are already highly significant. Continuing this study over time and expanding it spatially are among our objectives.

Third, only approximately 25% of taxa were resolved to the species level. Although this limitation is acknowledged, it is not adequately integrated into the interpretation of the results.

We agree with the reviewer, this point deserved to be explained more clearly. We have added the following sentences to the discussion:

“The relatively low percentage of MOTUs assigned to the species level in our results should nevertheless have a relatively limited impact on the conclusions. Future improvements in taxonomic assignments would not significantly alter the trends observed, for example, in taxonomic ß-diversity, particularly because the MOTUs shared across sites would remain the same. Phylogenetic diversity calculation could become more precise, but wi

Attachment Submitted filename: Response to Reviewers.docx

# Editor report: Decision Letter 1

**DOI:** 10.1371/journal.pone.0352249.r004

**Authors:** Petr Heneberg

9 Jun 2026

First eDNA-based monitoring of marine vertebrates in the urban marine ecosystem of Abu Dhabi: mangrove and seagrass beds as biodiversity reservoirs?

PONE-D-26-08592R1

Dear Dr. Jung,

We’re pleased to inform you that your manuscript has been judged scientifically suitable for publication and will be formally accepted for publication once it meets all outstanding technical requirements.

Within one week, you’ll receive an e-mail detailing the required amendments. When these have been addressed, you’ll receive a formal acceptance letter and your manuscript will be scheduled for publication.

An invoice will be generated when your article is formally accepted. Please note, if your institution has a publishing partnership with PLOS and your article meets the relevant criteria, all or part of your publication costs will be covered. Please make sure your user information is up-to-date by logging into Editorial Manager at Editorial Manager® and clicking the ‘Update My Information' link at the top of the page. For questions related to billing, please contact billing support.

If your institution or institutions have a press office, please notify them about your upcoming paper to help maximize its impact. If they’ll be preparing press materials, please inform our press team as soon as possible -- no later than 48 hours after receiving the formal acceptance. Your manuscript will remain under strict press embargo until 2 pm Eastern Time on the date of publication. For more information, please contact onepress@plos.org.

Kind regards,

Petr Heneberg

Academic Editor

PLOS One

Additional Editor Comments (optional):

Reviewers' comments:

# Editor report: Acceptance letter

**DOI:** 10.1371/journal.pone.0352249.r005

**Authors:** Petr Heneberg

PONE-D-26-08592R1

PLOS One

Dear Dr. Jung,

I'm pleased to inform you that your manuscript has been deemed suitable for publication in PLOS One. Congratulations! Your manuscript is now being handed over to our production team.

At this stage, our production department will prepare your paper for publication. This includes ensuring the following:

* All references, tables, and figures are properly cited

* All relevant supporting information is included in the manuscript submission,

* There are no issues that prevent the paper from being properly typeset

You will receive further instructions from the production team, including instructions on how to review your proof when it is ready. Please keep in mind that we are working through a large volume of accepted articles, so please give us a few days to review your paper and let you know the next and final steps.

Lastly, if your institution or institutions have a press office, please let them know about your upcoming paper now to help maximize its impact. If they'll be preparing press materials, please inform our press team within the next 48 hours. Your manuscript will remain under strict press embargo until 2 pm Eastern Time on the date of publication. For more information, please contact onepress@plos.org.

You will receive an invoice from PLOS for your publication fee after your manuscript has reached the completed accept phase. If you receive an email requesting payment before acceptance or for any other service, this may be a phishing scheme. Learn how to identify phishing emails and protect your accounts at https://explore.plos.org/phishing.

If we can help with anything else, please email us at customercare@plos.org.

Thank you for submitting your work to PLOS ONE and supporting open access.

Kind regards,

PLOS ONE Editorial Office Staff

on behalf of

Dr. Petr Heneberg

Academic Editor

PLOS One
